# Can fractal dimensions objectivize gastropod shell morphometrics? A case study from Lake Lugu (SW China)

**DOI:** 10.1002/ece3.8622

**Published:** 2022-03-01

**Authors:** Robert Wiese, Kyle Harrington, Kai Hartmann, Manja Hethke, Thomas von Rintelen, Hucai Zhang, Le‐Jia Zhang, Frank Riedel

**Affiliations:** ^1^ Institute of Geological Sciences Freie Universität Berlin Berlin Germany; ^2^ Virtual Technology & Design University of Idaho Moscow Idaho USA; ^3^ Image Data Analysis Max Delbrück Center for Molecular Medicine Berlin Germany; ^4^ Computational Sciences and Engineering Oak Ridge National Laboratory Oak Ridge Tennessee USA; ^5^ Institute of Geographical Sciences Freie Universität Berlin Berlin Germany; ^6^ Museum für Naturkunde Leibniz‐Institut für Evolutions‐ und Biodiversitätsforschung Berlin Germany; ^7^ 12635 Institute for Ecological Research and Pollution Control of Plateau Lakes School of Ecology and Environmental Science Yunnan University Kunming China

**Keywords:** 3D fractal dimensions, 3D models, Ancient lake, DNA sequencing, landmark analysis, morphometrics, non‐marine gastropods, Viviparidae

## Abstract

1. Morphometrics are fundamental for the analysis of size and shape in fossils, particularly because soft parts or DNA are rarely preserved and hard parts such as shells are commonly the only source of information. Geometric morphometrics, that is, landmark analysis, is well established for the description of shape but it exhibits a couple of shortcomings resulting from subjective choices during landmarking (number and position of landmarks) and from difficulties in resolving shape at the level of micro‐sculpture.
2. With the aid of high‐resolution 3D scanning technology and analyses of fractal dimensions, we test whether such shortcomings of linear and landmark morphometrics can be overcome. As a model group, we selected a clade of modern viviparid gastropods from Lake Lugu, with shells that show a high degree of sculptural variation. Linear and landmark analyses were applied to the same shells in order to establish the fractal dimensions. The genetic diversity of the gastropod clade was assessed.
3. The genetic results suggest that the gastropod clade represents a single species. The results of all morphometric methods applied are in line with the genetic results, which is that no specific morphotype could be delimited. Apart from this overall agreement, landmark and fractal dimension analyses do not correspond to each other but represent data sets with different information. Generally, the fractal dimension values quantify the roughness of the shell surface, the resolution of the 3D scans determining the level. In our approach, we captured the micro‐sculpture but not the first‐order sculptural elements, which explains that fractal dimension and landmark data are not in phase.
4. We can show that analyzing fractal dimensions of gastropod shells opens a window to more detailed information that can be considered in evolutionary and ecological contexts. We propose that using low‐resolution 3D scans may successfully substitute landmark analyses because it overcomes the subjective landmarking. Analyses of 3D scans with higher resolution than used in this study will provide surface roughness information at the mineralogical level. We suggest that fractal dimension analyses of a combination of differently resolved 3D models will significantly improve the quality of shell morphometrics.

## Graphical abstract

In our study, we compare established morphological and morphometrical methods to the new approach of fractal geometry with respect to a group of freshwater gastropods from Lake Lugu (China) as a model group. Our results show that fractal geometry can help to overcome shortcomings of these established methods, since it might help to objectivize shell morphologies.

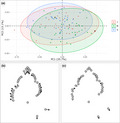

## 1 INTRODUCTION

The fossil record is crucial for studying morphological change and evolutionary patterns over long time intervals (Neubauer et al., 2013). Preservation of soft parts is uncommon and the reconstruction of ancient DNA is limited to Quaternary sedimentary archives (Shapiro et al., 2019; Stahlschmidt et al., 2019). Thus, evolutionary paleontologists preferentially focus on fossils in 3D preservation such as shells or bones which allow relatively complex morphological analyses within a conceptual framework termed constructional morphology (Thomas, 1979). Paleontology naturally interlinks with neontology through the usage of biological studies for paleontological purposes, often leading to ambiguous results because of conflicting character sets, such as molecular genetics, anatomy, and morphology (Becker et al., 2016; Stepanović et al., 2016). The causes of conflicting data are many sided. A major challenge is certainly how to properly describe physical reality (Einstein et al., 1935; MacLeod & Forey, 2002; Raup & Stanley, 1971). The quantitative study of (paleo‐) biological forms has developed from linear to geometric morphometrics (Adams et al., 2013), termed a “revolution in morphometrics” about 3 decades ago (Rohlf & Marcus, 1993). The selection of a morphometric technique depends on the shape and preservation of an object (Van Bocxlaer & Schultheiß, 2010) and on the researcher´s decisions about the number of landmarks or 2D or 3D approach to be applied, which may lead to significantly different results (Márquez & Averbuj, 2017; Tajika & Klug, 2020). Against this background, Porto and Voje (2020) recently proposed an approach for automated landmarking.

Reichert et al. (2017) emphasized “the power of 3D fractal dimensions” for comparing shapes in an objective way. Based on Mandelbrot (1982) and his concept of fractal geometry, another more secret “revolution in morphometrics” may pick up speed despite the criticism “that a *fractal cow* is often not much better than a *spherical cow*” (Buldyrev, 2012). Quite a few studies across (paleo‐) biological disciplines have demonstrated the potential of fractals for morphometrics (Aiello et al., 2007; Bruno et al., 2008; Isaeva et al., 2006; Klinkenbuß et al., 2020; Lutz & Boyajian, 1995). Kaczor et al. (2012) suggested fractal dimensions as an indicator of roughness in protein structures.

In our study, we apply 3D‐fractal‐ as well as 2D‐landmark morphometry to shells of freshwater gastropods. In general, gastropod shells represent the most diverse and abundant Cenozoic macrofossils (Allmon & Smith, 2011; Erwin & Signor, 1991; Morris & Taylor, 2000; Riedel, 2000). Non‐marine aquatic gastropods represent a smaller portion of the biodiversity, but still, several thousand modern species inhabit rivers, lakes, ponds, and wetlands worldwide (Strong et al., 2008). Neubauer et al. (2014) reported more than 2,000 valid taxa from the European Neogene alone. The morphometric challenge is emphasized by the fact that the morphological disparity in non‐marine aquatic gastropods is on average lower than in their marine counterparts (Riedel, 1993, 2000) but that phenotypic plasticity of shells is “at least three times larger” in freshwater species (Bourdeau et al., 2015).

We here focus on viviparid gastropods from Lake Lugu, located on the Yunnan‐Guizhou Plateau in southwestern China (Wiese et al., 2020). Viviparids have a Jurassic origin, and they dispersed to all continents except for Antarctica and South America (Van Bocxlaer & Strong, 2016). Southeast Asia has been identified as a biodiversity hotspot which is reflected by a greater morphological variability in the viviparid shells, particularly by more prominent sculptural elements (Stelbrink et al., 2020). Lake Lugu is considered a putative ancient lake with a high gastropod diversity including the three viviparid genera *Sinotaia*, *Cipangopaludina*, and *Margarya* (Wiese et al., 2020; Zhang et al., 2015). Du et al. (2012) also named the *Angulyagra* species *A*. *oxytropoides*, inhabiting the lake. The species *A*. *oytropoides* nowadays is referred as *Margarya oxytropoides* (Zhang et al., 2015) and therefore, we cannot exclude that Du et al. (2012) referred to the taxa, analyzed in this study. Another enigmatic species is the gastropod Valvata “luguensis,” which was mentioned by Du et al. (2017), but was not formally described or depicted. Shells of *Cipangopaludina* and *Margarya* have similar outlines and sizes, but sculptural elements are usually weak in *Cipangopaludina* and pronounced in *Margarya* (Van Bocxlaer & Strong, 2016; Zhang et al., 2015). Because of intermediate shell forms, Wiese et al. (2020) supposed that one to two species of each genus may inhabit Lake Lugu; however, neither genetic nor comprehensive morphometric studies were conducted and thus diversity and taxonomic assignments of the large Lake Lugu viviparids remain unresolved. The aim of this study is to morphometrically analyze *“Cipangopaludina*/*Margarya*” from diverse lake habitats of its two basins to test whether these results are in line with genetic and ecological data and to infer the value of fractal dimension analyses for the description of shape.

## 2 MATERIALS AND METHODS

### 2.1 Samples

**FIGURE 1:**
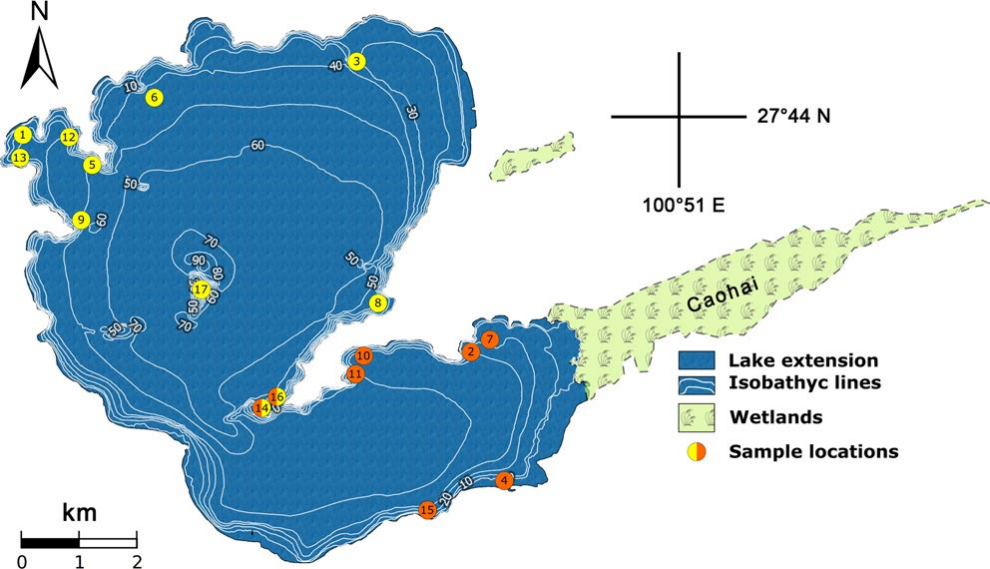
Lake Lugu sample locations of *“Cipangopaludina*/*Margarya”* (modified from Wiese et al., 2020). Yellow dots indicate locations in the northern basin, orange dots those from the southern basin, and yellow/orange ones mark the transitional zone

**FIGURE 2:**
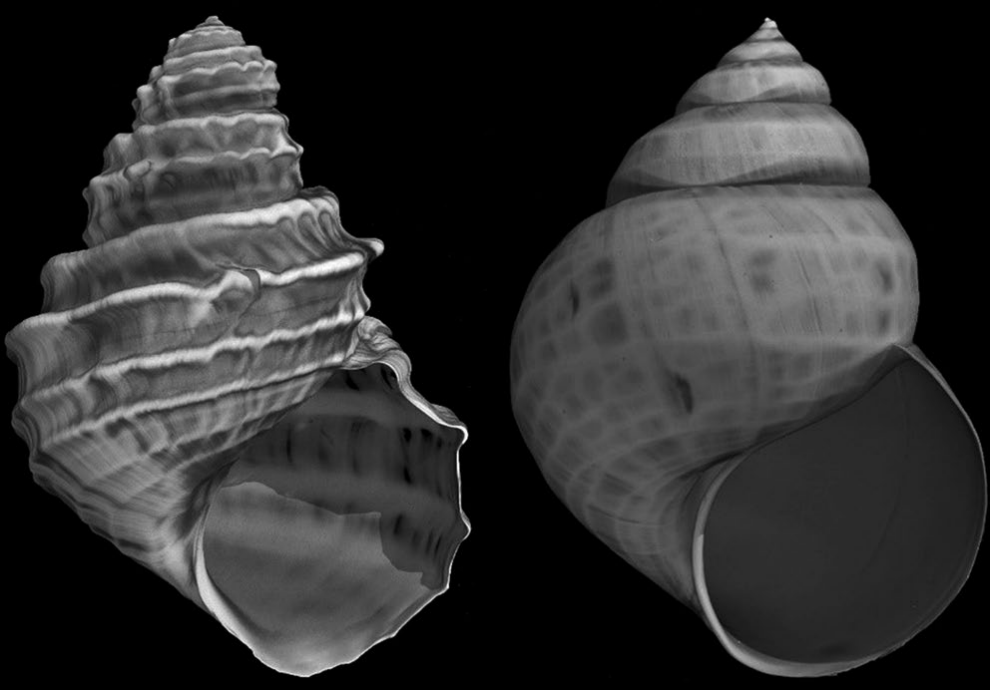
Tomograph images of the most strongly sculptured shell of this study, *Margarya melanioides* from Lake Dianchi (left) and a *Cipangopaludina* from Lake Erhai (right), both used as outgroups for the Lake Lugu shells (not to scale)

The Lake Lugu samples were obtained in September and October 2014. In the shallow littoral areas, “*Cipangopaludina/Margarya*” specimens were taken with a landing net. Samples from deeper water areas of up to 6 m depths were taken via snorkel diving. In total, 17 locations all over the lake were sampled (Figure 1; Table 1). The gastropods were preserved in 90% ethanol and are deposited at the Museum für Naturkunde Berlin (MfN, Germany) collection. *Cipangopaludina* sp. from Lake Erhai was sampled in October 2011 and *Margarya melanioides* from Lake Dianchi in April 2012 (one specimen of each species; see Table 1) (Figure 2).

**TABLE 1:** Sample locations; number of specimens (serial number) examined from corresponding location: 99 individuals in total of which 33 (in brackets) were studied genetically; MfN = Museum für Naturkunde Berlin. Due to intermediate forms, specimens from Lake Lugu were not assigned to a specific genus, but to the genera complex “*Cipangopaludina*/*Margarya*” to compare them with assured genus assignments (samples 98 and 99)

Locations (1–17 Lugu)	GPS coordinates	Specimens (COI sampled)	Collection ID (MfN)	Viviparid genus
1	N27°43′46″ E100°44′43″	1 (1)	113674	*“Cipangopaludina/Margarya”*
2	N27°41′45″ E100°48′59″	2–6 (4,6)	121321	*“Cipangopaludina/Margarya”*
3	N27°44′32″ E100°47′58″	7–13 (8–10)	121322	*“Cipangopaludina/Margarya”*
4	N27°40′31″ E100°49′22″	14–20	121323	*“Cipangopaludina/Margarya”*
5	N27°43′38″ E100°45′28″	21 (21)	121324	*“Cipangopaludina/Margarya”*
6	N27°44′15″ E100°45′56″	22–27 (22,24,26)	121330	*“Cipangopaludina/Margarya”*
7	N27°41′53″ E100°49′13″	28–30 (28)	121331	*“Cipangopaludina/Margarya”*
8	N27°42′10″ E100°48′17″	31–38 (31,37)	121335	*“Cipangopaludina/Margarya”*
9	N27°42′55″ E100°45′20″	39	121337	*“Cipangopaludina/Margarya”*
10	N27°41′49″ E100°47′55″	40–45 (40,45)	121338	*“Cipangopaludina/Margarya”*
11	N27°41′45″ E100°47′49″	46–49 (46)	121340	*“Cipangopaludina/Margarya”*
12	N27°43″52″ E100°45″24″	50–57 (55)	121341	*“Cipangopaludina/Margarya”*
13	N27°43′50″ E100°44′44″	58–68 (62,63,67)	121342	*“Cipangopaludina/Margarya”*
14	N27°41′13″ E100°47′03″	69–72 (70,71,72)	121344	*“Cipangopaludina/Margarya”*
15	N27°40′11″ E100°48′31″	73–79	127438	*“Cipangopaludina/Margarya”*
16	N27°41′15″ E100°47′08″	80–91 (82,83,85,88,91)	121328 a/b	*“Cipangopaludina/Margarya”*
17	N27°42′24″ E100°46′30″	92–97 (92–95,97)	121329 a/b	*“Cipangopaludina/Margarya”*
Lake Erhai	N25°41′08″ E100°16′13″	98	Gast_Viv_Erh_1	*Cipangopaludina*
Lake Dianchi	N24°43′49″ E102°39′21″	99	Gast_Viv_Dia_1‐	*Margarya*

### 2.2 General shell morphological analyses

**FIGURE 3:**
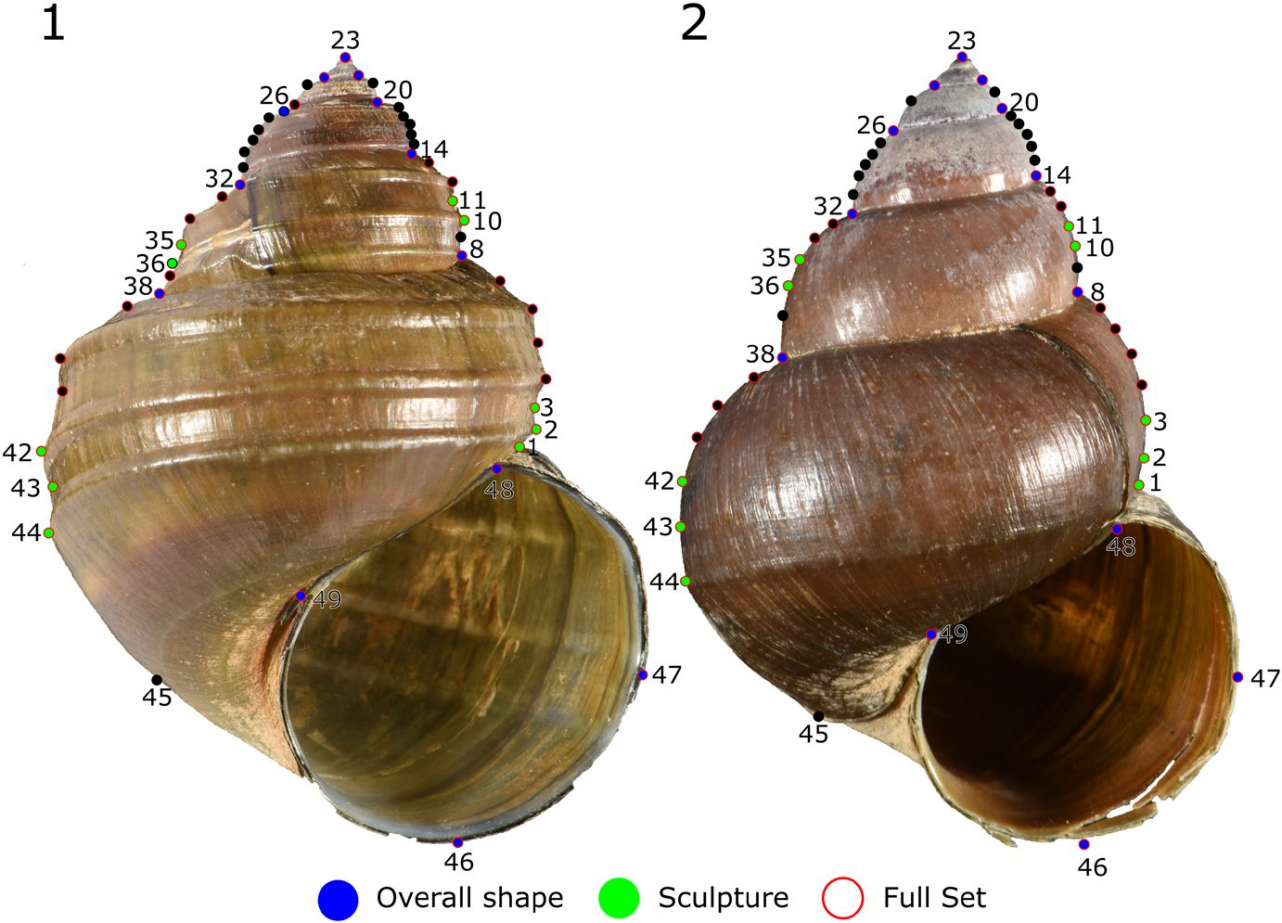
Landmarking of a strongly (1) and a weakly keeled shell (2). Total number of landmarks per shell is 49. Subsets are represented by blue dots (overall shape), green dots (sculpture), and red outlines (full set)

**FIGURE 4:**
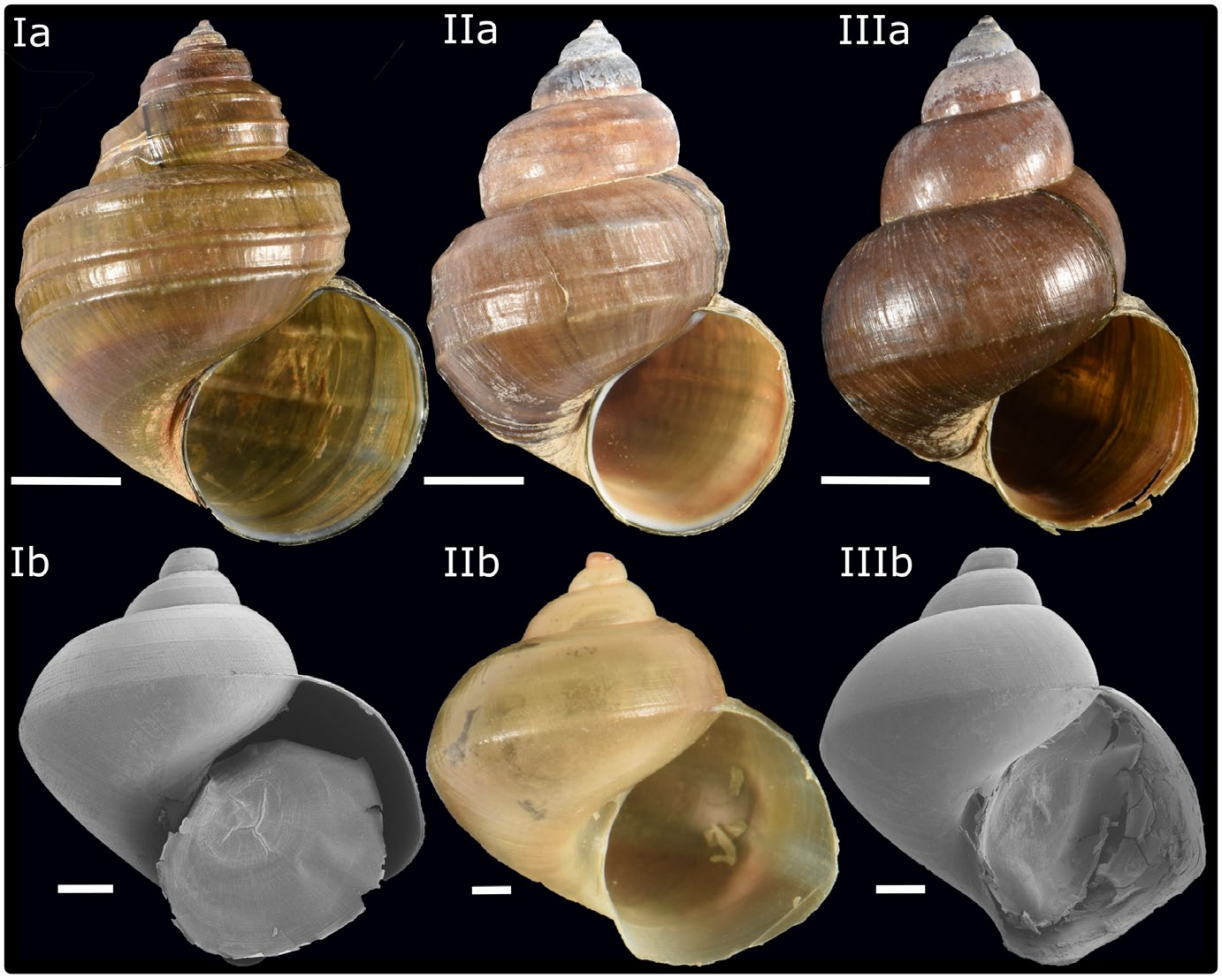
General morphotypes of Lake Lugu *“Cipangopaludina*/*Margarya*.*”* Upper row: Examples of strongly (Ia), intermediately (IIa), and weakly sculptured adult shells (IIIa). Lower row: Embryonic shells (Ib‐IIIb) retrieved from female specimens assigned to the three different sculptural categories (Ia‐IIIa) show no significant differences. Scales represent 1 cm (upper row) and 1 mm (lower row)

Shells of 99 mostly full‐grown individuals (Table 1) were equally aligned (Figure 3) with preparation dough, when documented with a Nikon D300 camera. We considered specimens, significantly smaller than the average shells as not fully grown. The photographs were used to measure several morphological features, namely maximum height and maximum width, height and width of the aperture, and the height of the spira. Eventually, we calculated the height/width ratio, the height/width ratio of the aperture, the height spira/height shell ratio, and the height aperture/height shell ratio. Macroscopic analysis in the field revealed that sculpture varies from weakly (“*Cipangopaludina”*) to strongly pronounced (*“Margarya”*) with no obvious clustering at the terminal ranges. Rather intermediate forms exist which could not clearly be assigned to either the *“Cipangopaludina”* or to the *“Margarya”* type. In order to test whether mathematical and visual analyses are basically in line, each of the shells was assigned to one of three subjective sculpture categories: strong (Figure 4Ia), intermediate (Figure 4IIa), and weak (Figure 4IIIa). These categories refer to the first‐order sculpture (here: spiral keels). Second‐order sculpture such as growth increments or minute lirae is not addressed with these terms. Selected early ontogenetic shells were retrieved from the ovoviviparous females (Riedel, 1993) and studied under a Zeiss scanning electron microscope.

The original research design focused on first‐order sculpture and therefore, the sample selection was not based on well‐preserved second‐order sculpture. However, in the course of our study, we additionally investigated the second‐order sculpture of 12 specimens using a scanning electron microscope and the 3D scans to capture less obvious differences.

### 2.3 Fractal dimensions of shells using the program SnailJ

The 99 shells were 3D scanned with a Phoenix Nanotom S X‐ray tomograph (µCT) at the Micro CT Lab of the Museum für Naturkunde Berlin. Each shell was X‐rayed in 0.25° angular distances with a total of 1440 scans. The scans were computed to a 3D model with *datos |x 2*.*0*; surface meshes were exported as STL files for further image processing.

The *SnailJ* plugin was developed for this study to conduct fractal analysis with the Fiji distribution (Schindelin et al., 2012) of *ImageJ* (Schneider et al., 2012). *SnailJ* proceeds by first voxelizing. STL meshes were at a user‐defined pixel resolution, here 500(x), 500(y), and 500(z). Voxelization transforms surface mesh data into a 3D image represented in Cartesian space required for box counting. The box counting algorithm is then applied to calculate the fractal dimension or Minkowski–Bouligand dimension *D*_MB_ (Doube et al., 2010; Mandelbrot, 1982; Parkinson & Fazzalari, 2000).

Due to the limited statistical function of *SnailJ*, fractal analyses were conducted in R. Fractal dimensions, here termed Minkowski–Bouligand (MB) dimensions, and the 95% confidence bounces were calculated via box counting. Densities of MB dimensions and standard errors were calculated and plotted to analyze the distribution of all measured values. This was done simultaneously for the three sculpture categories. For sculpture categories “weak” and “strong,” the residuals of the fractals were plotted. Calculations were eventually repeated with smaller box sizes.

### 2.4 Landmark analyses of shells

Landmarks were set with the *ImageJ Point Picker* plugin (Thévenaz, 2010). In total, 49 landmarks per shell were identified to potentially reflect shape including sculpture (Figure 3). Of these 49 homologous points, three data sets of landmarks evolved, which were used for further analyses. A total of 34 of these landmarks were chosen to represent the full set of morphology (Table 2). Ten homologous landmarks were chosen to evaluate sculpture (green dots in Figure 3, Table 3) and thirteen were chosen to characterize overall shape (blue dots in Figure 3, Table 2). The full set of landmarks was used to detect possible differences between shells from the northern and southern basins of Lake Lugu (see Figure 1).

**TABLE 2:** Data subsets and the landmarks, which were used for the analyses

Data subset	Landmarks used
Overall shape	8, 14, 20, 22, 23, 24, 26, 32, 38, 46, 47, 48, 49
Sculpture	1, 2, 3, 10, 11, 35, 36, 42, 43, 44
Full set	1, 2, 3, 4, 5, 6, 7, 8, 10, 11, 12, 13, 14, 20, 22, 23, 24, 26, 32, 33, 34, 35, 36, 38, 39, 40, 41, 42, 43, 44, 46, 47, 48, 49

**TABLE 3:** Explanation of homologous landmarks for sculpture data set

Landmark	Homology
1	Suture between body whorl and spire
2	Highest point of first keel on body whorl (right)
3	Lowest point between first and second keel on body whorl (right)
10	Highest point on first keel of first spire whorl (right)
11	Lowest point between first and second keel on second spire whorl (right)
35	Lowest point between first and second keel on second spire whorl (left)
36	Highest point on first keel of first spire whorl (left)
42	Highest point of second keel on body whorl (left)
43	Lowest point between first and second keel on body whorl (left)
44	Highest point of first keel on body whorl (left)

Landmark coordinate outputs from the *ImageJ Point Picker* plugin (Table S1) were provided with an identifier (sample number from the MfN collection) and information on the number of landmarks. Landmark analyses were conducted in R (R Core Team, 2020), in parts following a routine outlined by Theska et al. (2020). Generalized procrustes analysis (GPA) of the two‐dimensional, fixed‐landmark coordinates was performed using function gpagen{geomorph} (Adams et al., 2013). Procrustes shape variables were then analyzed using principal component analysis (PCA). Further statistical testing, which assessed whether north and south basin gastropods were morphologically distinct, included procrustes ANOVA based on Euclidean distances using function procD.lm{geomorph}. The number of iterations for significance testing was set to 100,000. *p*‐values were adjusted for false discoveries among the rejected hypotheses using p.adjust{stats}, method “fdr.” All landmark analyses are documented in detail in Data S1 and S2.

### 2.5 Genetic analyses

A subset of 33 *“Cipangopaludina*/*Margarya”* specimens from Lake Lugu (Table 1) was used for basic genetic analyses by sequencing the mitochondrial COI gene. Partial sequences of the mitochondrial cytochrome c oxidase subunit I (COI) gene were amplified through polymerase chain reaction (PCR) using primers LCO1490, 5′‐GGTCAACAAATCATAAAGATATTGG‐3′ (Folmer et al., 1994) and HCO2198var, 5′‐TAWACTTCTGGGTGKCCAAARAAT‐3′ (von Rintelen et al., 2004). PCR amplifications were conducted in volumes of 25 μl with an initial denaturing step at 94 °C for 3 min, followed by 35 cycles of 94°C for 30 s, 45°C for 1 min, and 72°C for 1 min, with a final extension step of 5 min at 72°C. Purification and cycle sequencing were conducted by Macrogen Europe.

The 33 DNA sequences were uploaded into GenBank (accession numbers and museum voucher numbers in Table S2). Nine additional sequences from other East and Southeast Asian viviparid species, from Stelbrink et al., 2020, were included in the analysis (Table S2).

The sequences were aligned using the *Muscle* algorithm (Edgar, 2004) as implemented in *Geneious Prime 2020* (https://www.geneious.com). The alignment was checked and adjusted manually. The genetic distances were calculated using *MEGA X* (Kumar et al., 2018). The data set was tested in *MEGA X* for the best‐fit model of sequence evolution by means of the Akaike and Bayesian information criteria. GTR+G was suggested as the best‐fitting nucleotide substitution model. Maximum likelihood (ML) analysis was conducted using *PhyML 3*.*3* (Guindon et al., 2010) implemented in *Geneious Prime 2020*. A total of 1,000 replicates were calculated to obtain bootstrap values. Bayesian inference (BI) was conducted using *MrBayes 3*.*2*.*6* (Ronquist et al., 2012) implemented in *Geneious Prime 2020* with four independent chains for 5,000,000 generations, samplefreq = 1,000, and burnin = 25%.

## 3 RESULTS

### 3.1 General shell morphology

According to Lu et al. (2014) and Zhang et al. (2015), the weakly sculptured large viviparids from Lake Lugu are *Cipangopaludina* and the strongly sculptured shells represent *Margarya*. The specimens from Lake Lugu, however, do not only represent these two morphotypes but exhibit a range of intermediate ones (see Section 2.2). Note that the intermediate shell type, displayed in Figure 4 (IIa), is only a representative example for a range of intermediate morphologies. We decided to subjectively assign the shells visually to three first‐order shell sculpture categories, which resulted in 32 strongly, 31 intermediately, and 36 weakly sculptured shells (Table S3).

**FIGURE 5:**
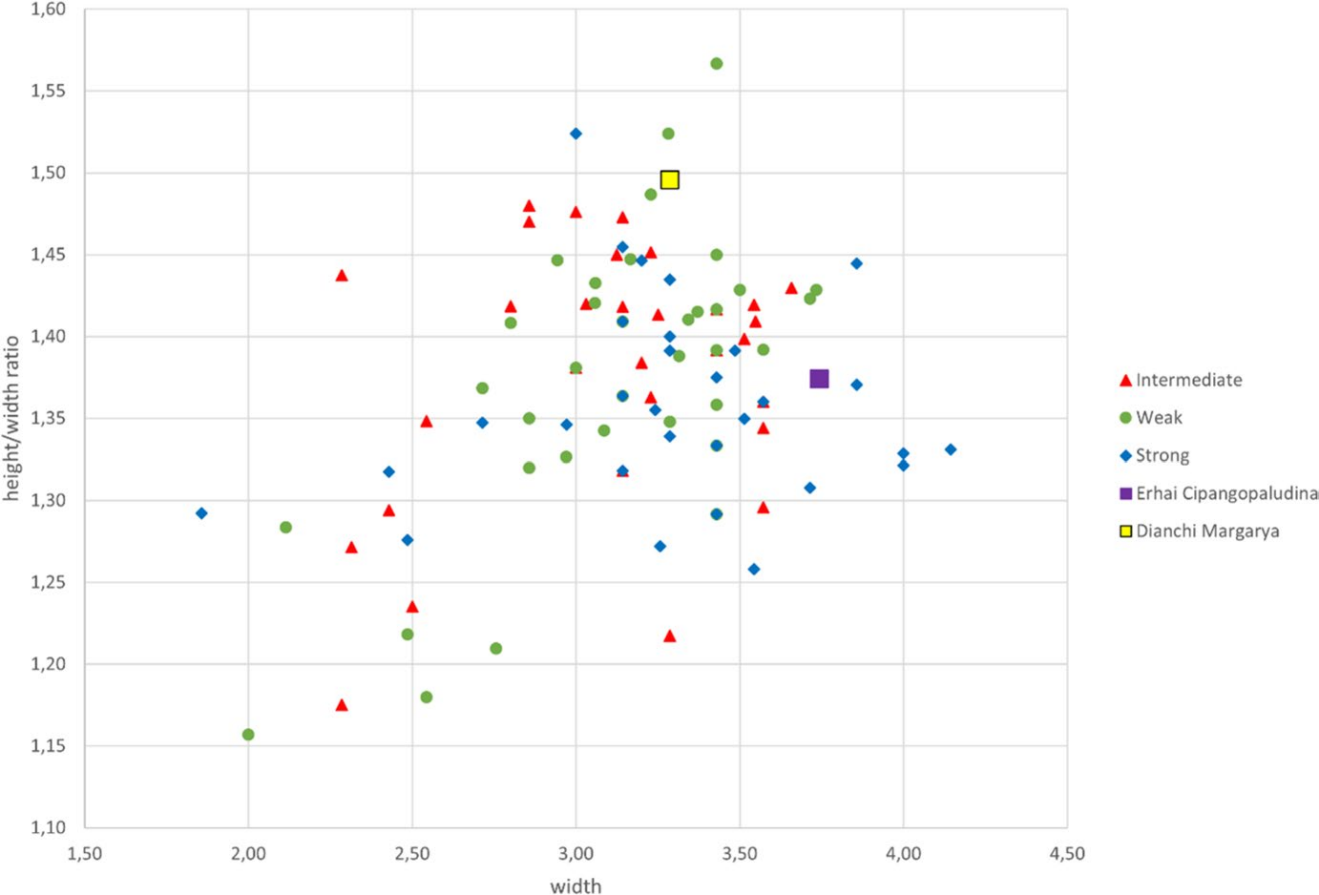
Height–width ratio versus width of *“Cipangopaludina*/*Margarya”* shells from Lake Lugu plus the outgroups Lake Dianchi *Margarya* which is strongly sculptured and Lake Erhai *Cipangopaludina* which is weakly sculptured. The three sculpture categories do not cluster

The maximum shell width ranges from 1.86 to 4.14 cm, the maximum height from 2.31 to 5.57 cm, and the height/width ratio from 1.16 to 1.57. Height–width dimensions do not correlate with sculptural categories (Figure 5). Aperture height varies between 1.19 cm and 2.76 cm, the aperture width between 1.08 cm and 2.25 cm. The height/width ratio of the aperture ranges from 0.97 to 1.35. The minimum spira height amounts to 1.15 cm, and the maximum spira height up to 3.55 cm. The ratio between spira height and shell height varies between 0.45 and 0.73, and the ratio between aperture height and shell height between 0.35 and 0.61. None of these features correlate with sculpture or would justify a taxonomic separation between these groups (Figures [Link], [Link], [Link]). Morphological data are summarized in Table S4.

The oldest embryos from females belonging to one of the three sculptural categories were documented and do not exhibit significant differences but are rather uniform. Embryonic shells are around 6.6 mm high and 6.3–6.6 mm wide (Figure 4Ib, Iib and IIIb).

### 3.2 Fractal analyses

Minkowski–Bouligand dimensions of the 99 shells vary in means within min_DMB_ = 2.059 and max_DMB_ = 2.246, with a mean value of *D*_MB_ = 2.166 (CI_95%_ = [2.025, 2.265]) and hence, cover a range of 0.187 (Table 4). The standard error (SE_D_) of all *D*_MB_ estimations vary in a range between min_SED_ = 0.007 and max_SED_ = 0.017. We, therefore, consider the measured differences (Table S5) as significant.

**TABLE 4:** Minkowski–Bouligand values: maximum, minimum, and ranges for sculpture categories and for all shells

Sample set	*D*_MB_max_	*D*_MB_min_	Range
Strong sculpture	2.187	2.059	0.128
Intermediate sculpture	2.210	2.112	0.098
Weak sculpture	2.246	2.114	0.132
All shells	2.246	2.059	0.187

**FIGURE 6:**
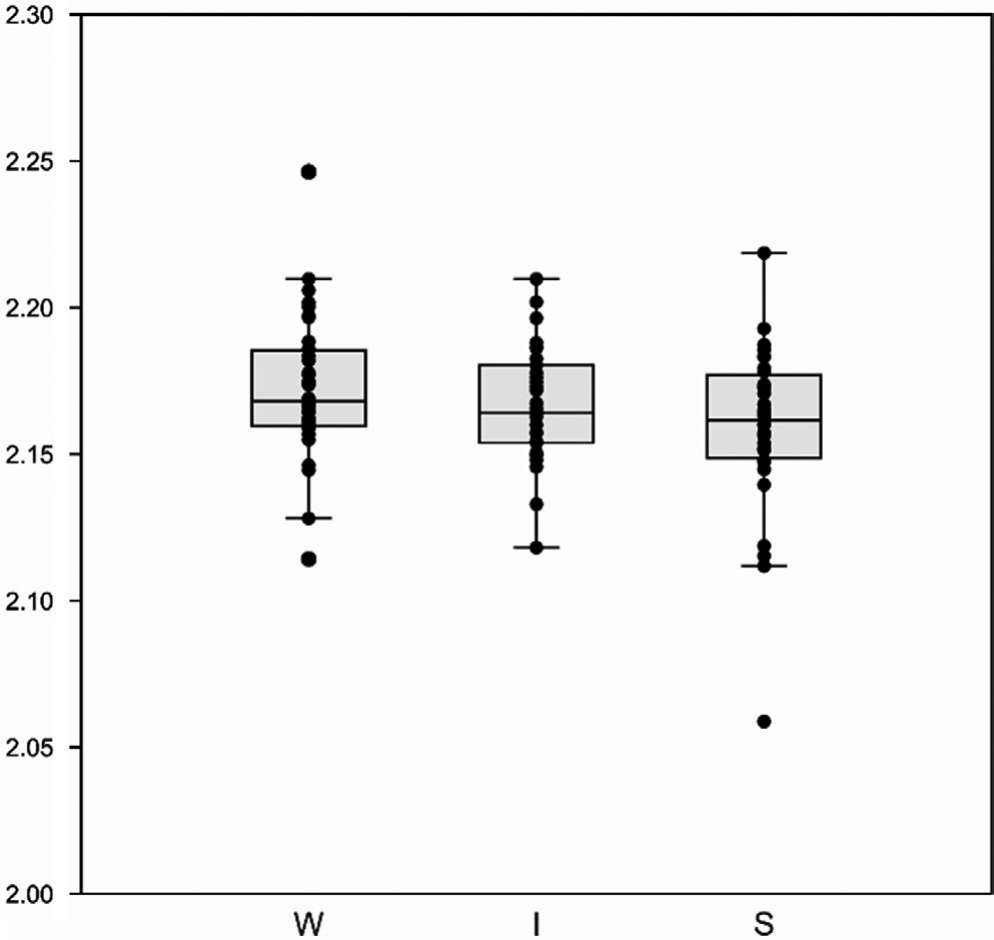
First‐order sculptural categories W = weak, I = intermediate, S = strong, plotted against *D*_MB_ values. Minkowski–Bouligand dimensions of the three categories cannot be statistically distinguished (ANOVA *p* = .07824; Tukey's pairwise *p* > .0625 for all three pairs)

In general, *D*_MB_ values are neither in agreement with size (max. shell height; Figure S2) nor with the three sculpture categories (Figure 6). The five highest values (ascending to max.) represent sculpture categories Weak/Intermediate/Weak/Strong/Weak sculpture and the five lowest values (descending to min.) represent sculpture categories Intermediate/Strong/Weak/Strong/Strong (Table S5).

**FIGURE 7:**
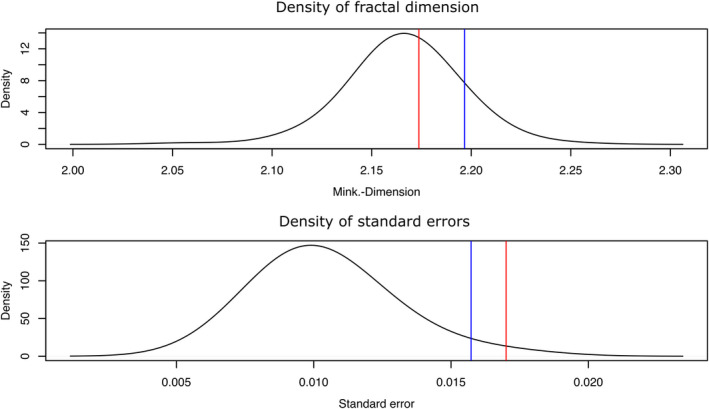
Density against fractal dimension (upper graph) and standard errors (lower graph) with a smoothing bandwidth of 0.02 of all scanned shells. Both distributions resemble normal distributions. Red lines mark the positions of the Lake Dianchi *Margarya*, blue lines those of the Lake Erhai *Cipangopaludina* (Figure 2)

With a box size down to 0.055 cm, densities of fractal dimensions and standard error almost plot within a normal distribution (Figure 7). The value of the Lake Dianchi *Margarya*, with the strongest sculpture of all shells (Figure 2), is not significantly higher than the overall mean (*p* < .38). However, its standard error is significantly higher (*p* < .0008) than the rest of the data set.

**FIGURE 8:**
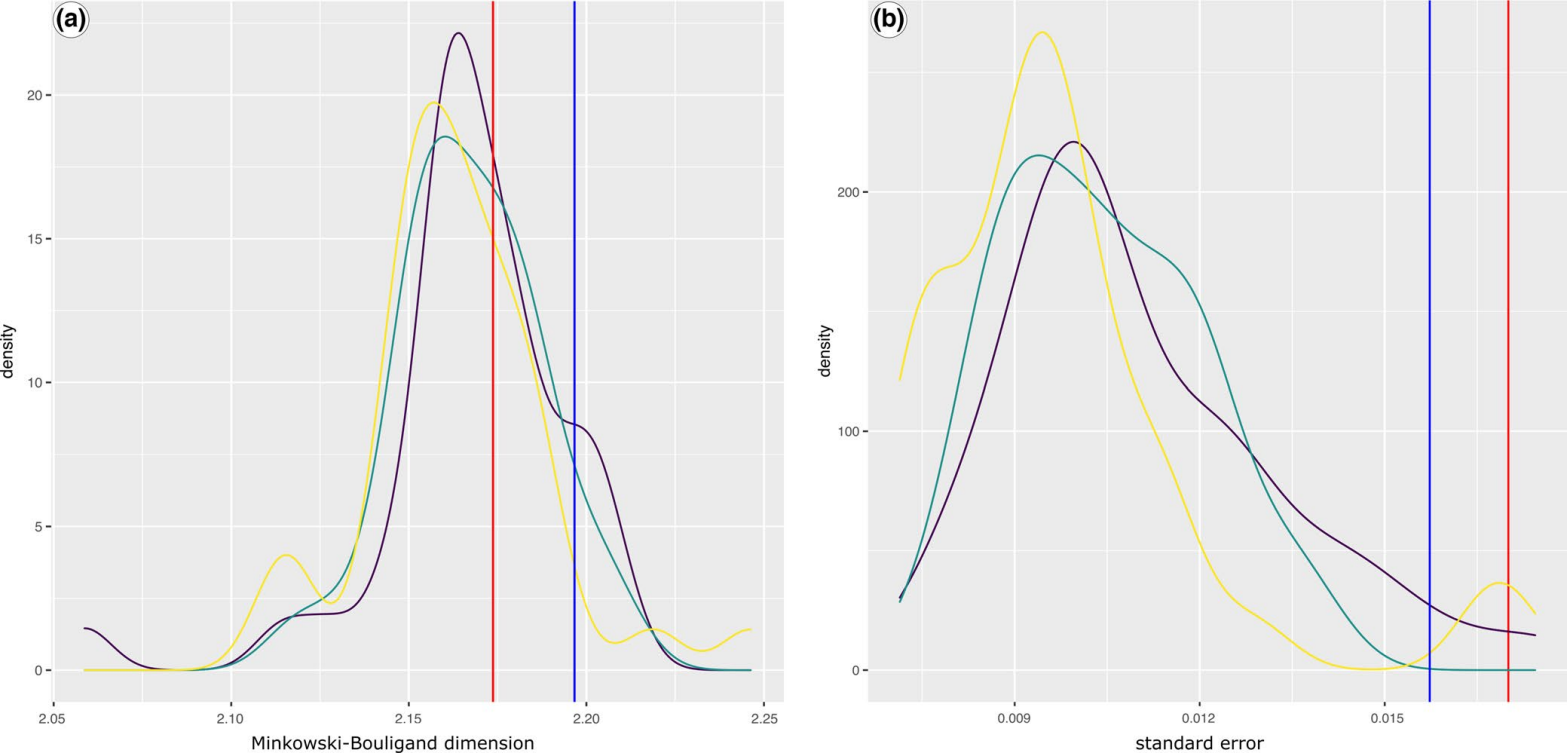
Density versus fractal dimension (a) and standard errors (b) of the different sculpture levels *with a box size larger than 0*.*055 cm*. The three different sculptural categories are represented by only slightly differing *D*_MB_ values (strong = yellow, intermediate = green, and weak = violet line). Red lines mark the Lake Dianchi *Margarya*, blue lines the Lake Erhai *Cipangopaludina* (Figure 2)

The *D*_MB_ value of the Lake Dianchi *Margarya* plots within the range of the strongly sculptured shells (Figure 8a), but yields the highest standard error of all sampled specimens (Figure 8b).

**FIGURE 9:**
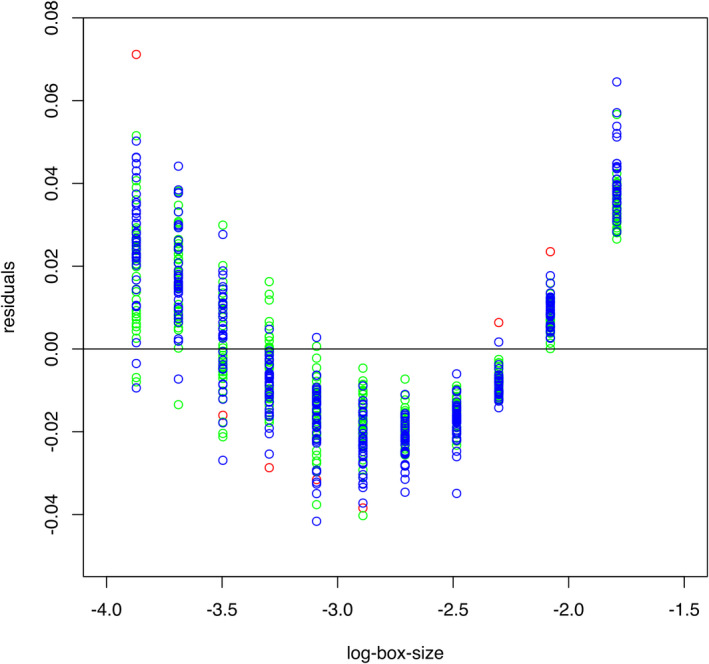
Residuals of strongly (green dots) and weakly sculptured (blue dots) shells in comparison with *Margarya* (red dots). The residuals within each box size are approximately normally distributed, but with a parabolic trend between the distinct box sizes

Testing residuals along box sizes, an obvious parabolic trend remains with a minimum at 0.055 cm (Figure 9; log box size (0.055 cm) = 2.89). Thus, we applied a *D*_MB_ estimation for box sizes smaller than 0.055 cm.

**FIGURE 10:**
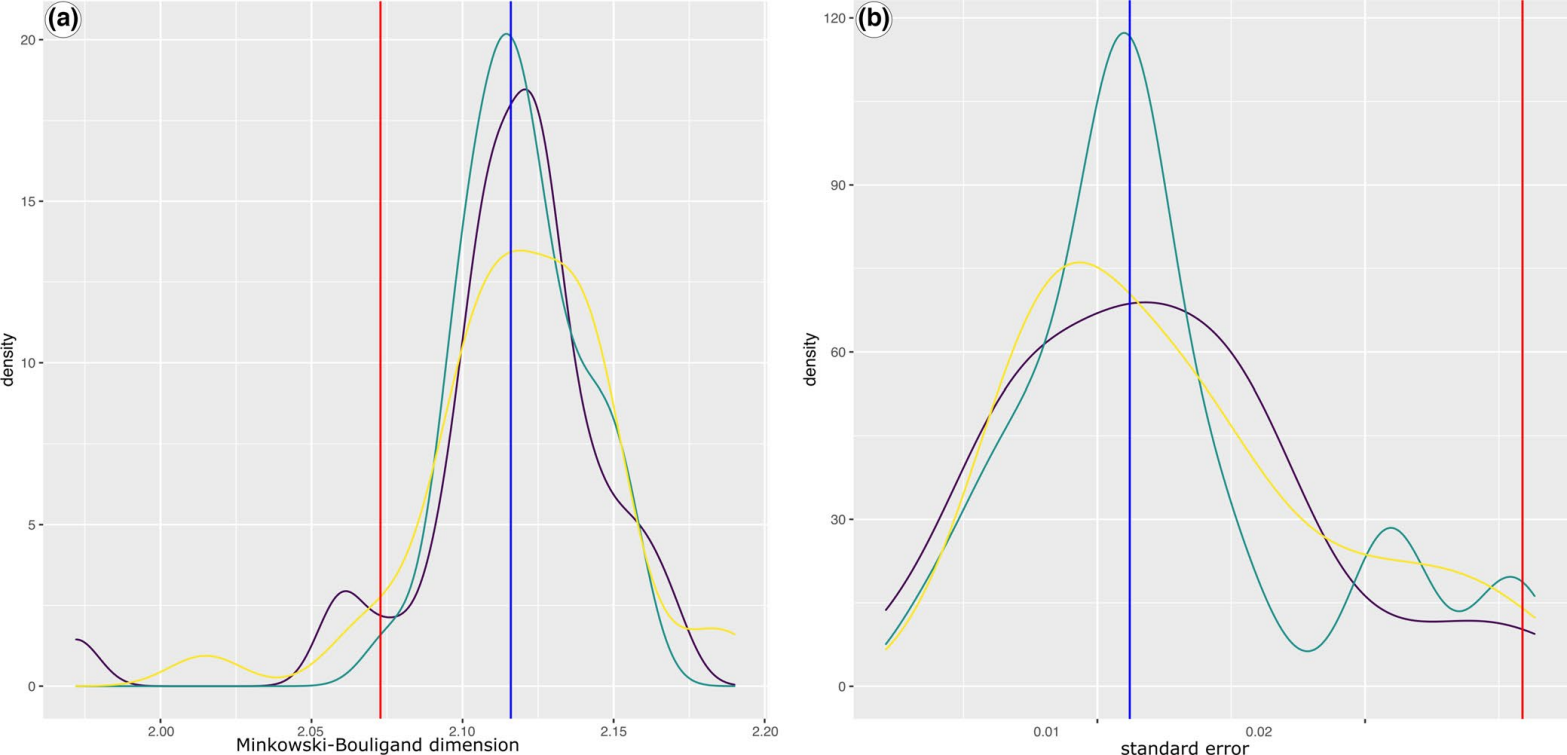
Density versus fractal dimension (a) and standard errors (b) of the different sculpture levels *with box sizes smaller than 0*.*055 cm*. *D*_MB_ values are similarly distributed in all three sculptural categories

Shrinking the box sizes results in normal distributions for the *D*_MB_ values for all three sculpture categories with almost the same positions (Figure 10a). Lake Dianchi *Margarya* is displayed in the lower tail area, indicating a significant lower fractal dimension than the majority of Lake Lugu *“Cipangopaludina*/*Margarya*.*”* The standard error distribution remains in the same range (Figure 10b) as for the larger box sizes (Figure 8b).

**FIGURE 11:**
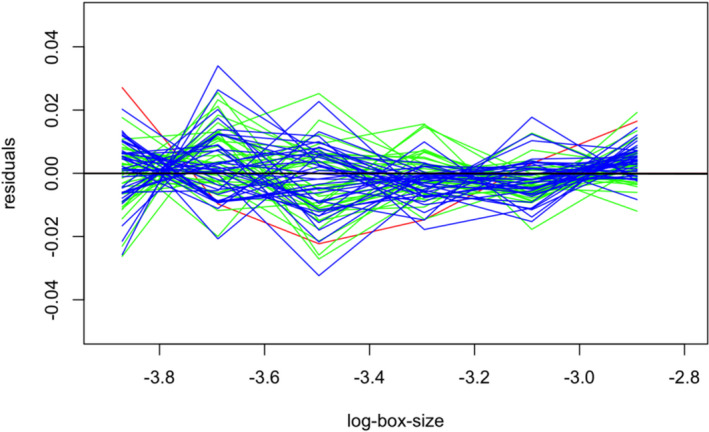
With smaller box sizes (Figure 10), the residuals for strongly (green) and weakly (blue) sculptured specimens are randomly distributed and stationary. The red line represents the residuals of the single *Margarya* shell with a remaining but slighter parabolic trend

With respect to the Lake Dianchi *Margarya*, the residuals appear random and stationary along the smaller box sizes (Figure 11).

**FIGURE 12:**
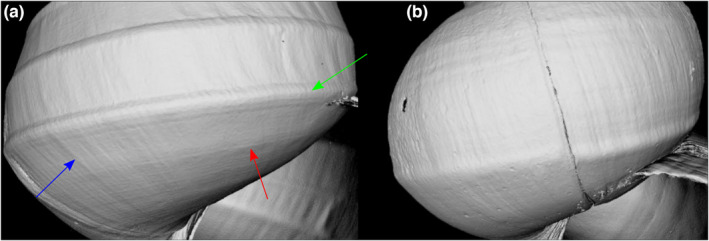
First‐order (spiral keels, green arrow) and second‐order sculpture: growth increments (blue arrow) and spiral lirae (red arrow) of the shells, exhibiting the most extreme Minkowski–Bouligand value *D*_MBmax_ value (a– 2.246) and *D*_MBmin_ value (b – 2.059). Specimen heights amount to 4.6 cm (a) and 3.0 cm (b)

Finally, the second‐order sculpture was checked under a microscope, and the shell with the highest *D*_MB_ value exhibits the highest number of spiral lirae (Figure 12a) while that with the lowest *D*_MB_ value (Figure 12b) is almost devoid of secondary spiral sculpture. One dozen of the shells were checked respectively and second‐order sculpture and *D*_MB_ values are in line. The number of spiral lirae is independent from the spiral keels (first‐order sculpture).

### 3.3 Landmark analyses

Landmark analyses of the sculpture set and the full set (see Section 2.4) both identify the strongly sculptured Lake Dianchi *Margarya* as the biggest outlier when assessing procrustes distances from the mean (Data S1.4.4 and S1.5.4), while it cannot be separated using the overall shape subset. To retain overall shape information, we chose to base further statistical analyses on the full set.

Principal component analysis of the procrustes shape variables for the full set resulted in three meaningful principal components (PC) that explain 35.7%, 13.1% and 8.4% of the variation, respectively. Shape change along PC1‐3 represents the height–width ratio, shell sculpture, and the ratio between body whorl and spire, respectively. Negative values of PC1 represent low height/width ratios and, therefore, compact, rather roundish shells. Positive PC1 values display high height/width ratios, representing elongated shells. Shells with strongly pronounced sculpture show positive PC2 values, whereas shells with weak sculpture tend to have negative PC2 values. Negative values of PC3 reflect higher spires than positive values.

**FIGURE 13:**
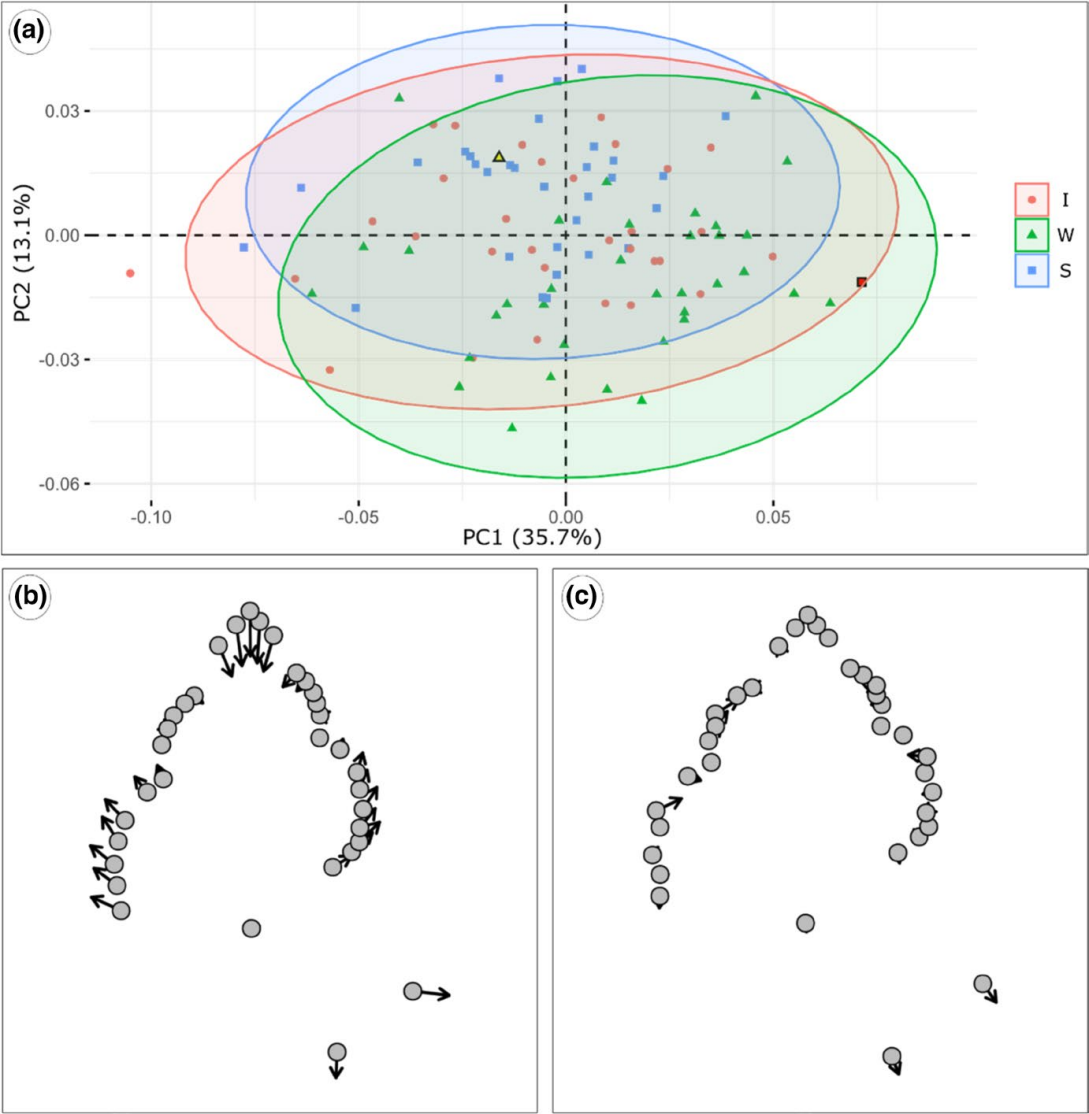
(a) Principal component analyses of the procrustes shape variables. The red square represents the strongly sculptured Lake Dianchi *Margarya*, and the yellow triangle the weakly sculptured Lake Erhai *Cipangopaludina*. (b), (c): Lollipop plots indicating the variation along PC1 (b) and PC2 (c), representing changes in the height–width ratio and shell sculpture, respectively

Generally, the three sculpture categories strongly overlap in shape space (Figure 13a). The shape space of the shells of genetically analyzed specimens (see Section 3.4) cannot be distinguished from the other shells (Figure S1).

**FIGURE 14:**
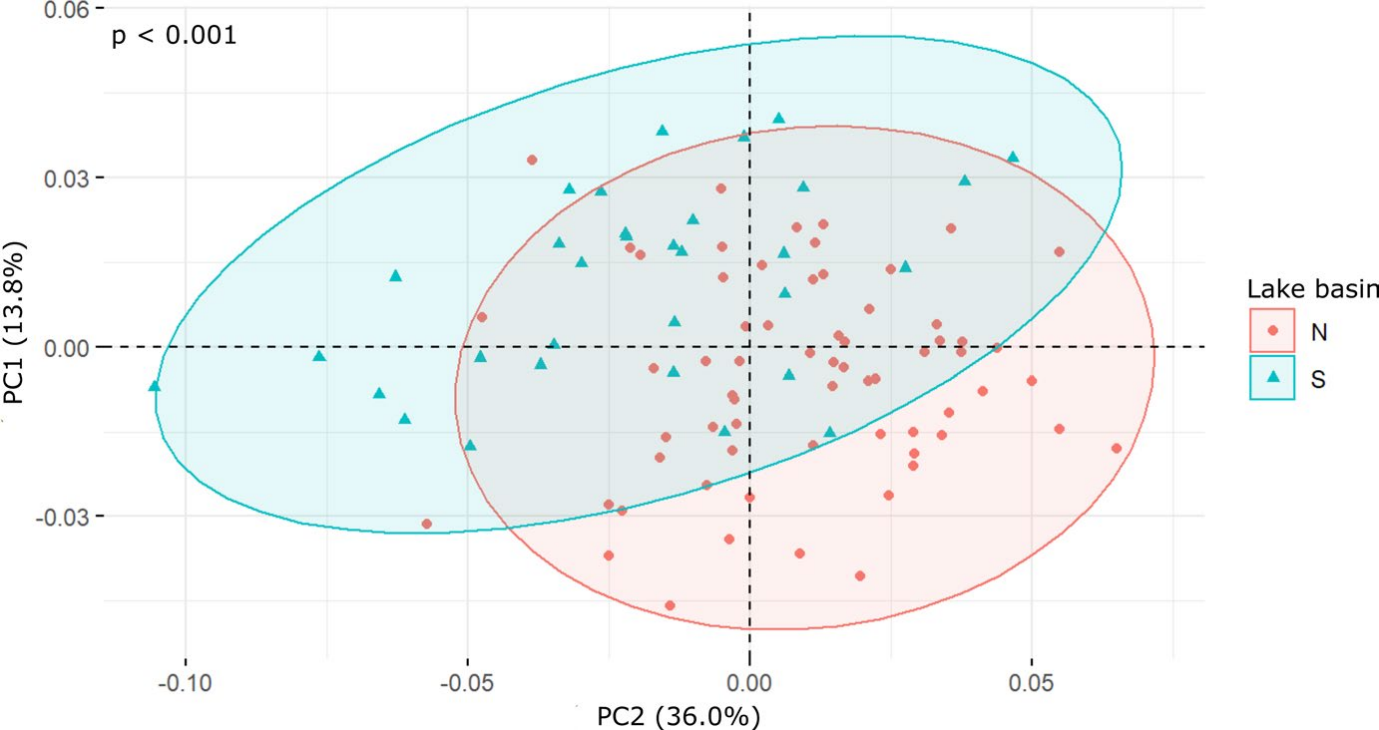
Principal component analyses of the procrustes shape variables of specimens from the northern (N) and the southern (S) basin (Figure 1), which are morphologically distinct at *p*_adj < .001

Landmark analysis of the full set indicates a large overlap between gastropod shape space of specimens from the northern and the southern lake basin. Yet, the two cohorts are morphologically distinct (Figure 14; *p* < .001, Data S2).

### 3.4 Genetic analyses

**FIGURE 15:**
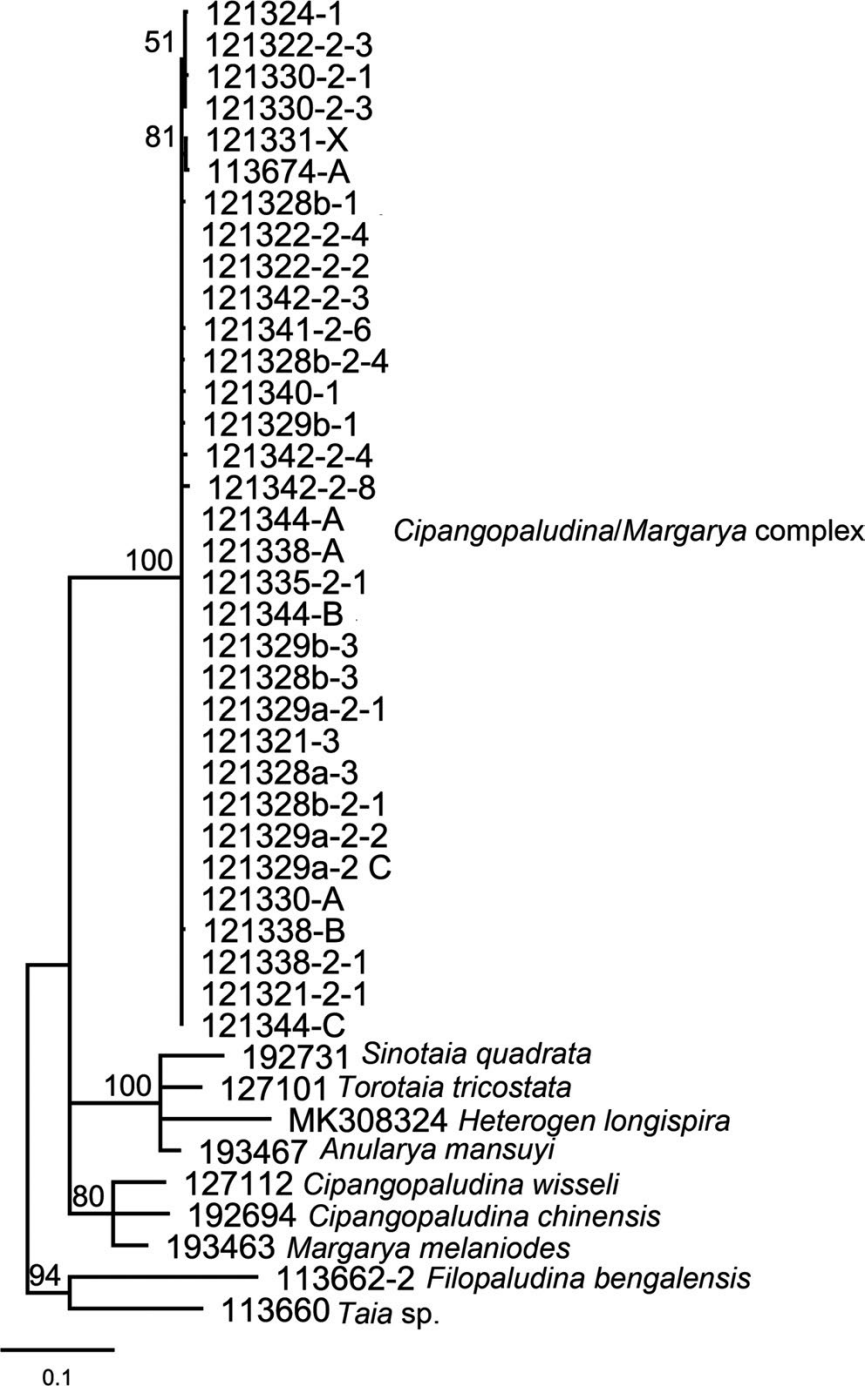
The Maximum likelihood tree for the *“Cipangopaludina*/*Margarya”* complex from Lake Lugu and other viviparid species of the subfamily Bellamyinae. Numbers above branches are bootstrap values. Bootstrap values below 50% are not shown

Landmark analysis revealed that the shells of the DNA analyzed specimens are representative of the morphological variation in the total set (Figure S1). Here, we only use the ML tree for illustration (Figure 15) since the BI tree has a similar topology. The ML tree demonstrates that the sequenced *“Cipangopaludina*/*Margarya”* from Lake Lugu form a monophyletic group (bootstrap value 100) distinct from other viviparids (Bellamyinae) included in this study. The split of the *“Cipangopaludina*/*Margarya”* complex into different species was not supported based on comb‐shape phylogeny and the short p‐distances within the complex (0%–0.47%).

## 4 DISCUSSION

In the title, we ask whether fractal dimensions can objectivize gastropod shell morphometrics. Against which background? How did our case study develop? We compared fractal dimensionality to traditional, established methods to evaluate its utility. Lake Lugu, a putative ancient lake, comprises a gastropod clade with highly variable shell morphologies which appear to us to represent a perfect model group to test the method.

So far the gastropod diversity of Lake Lugu has been mainly inferred conchologically. Only one genetic study of the genus *Radix* has recently been conducted (Wiese et al., 2020). Regarding the largest and thus most prominent gastropods of Lake Lugu, the viviparids, Wiese et al. (2020) listed the genera *Margarya*, *Cipangopaludina*, and *Sinotaia*. These gastropods can be found living also in other lakes of SW China such as Lake Erhai or Lake Dianchi (Zhang et al., 2015; personal observations). *Sinotaia* is not part of this study, as it is significantly smaller than the other two genera which exhibit a similar size range.

It is noteworthy that Lake Lugu represents the highest elevated ecosystem in which viviparids occur at all (Stelbrink et al., 2020; Wiese et al., 2020). There is some evidence that freshwater gastropods show extraordinary shell phenotypes under extreme environmental conditions (Clewing et al., 2015) and “shell shape variability is a critical factor in regional adaption” (Cazenave & Zanatta, 2016), but here we explicitly do not or only very briefly discuss the “contribution of genetic and environmental factors to shell shape variation” (Urabe, 1998). Our field observations in Lake Lugu, e.g., revealed that fish are cracking *“Cipangopaludina*/*Margarya”* shells independent of their sculpture. Sculpture is commonly related to predator avoidance (Covich, 2010). We also observed that *“Cipangopaludina*/*Margarya”* shells were covered by different degrees of algal growth (see Section 4.5). Shell‐attached algae are considered to contribute to the growth of the host *Cipangopaludina chinensis* (Fujibayashi et al., 2016). The scope of the following discussion, however, is primarily not to relate shell phenotypes with environmental parameters but to test the value of fractal dimension analyses for the description of shell shape, in comparison with traditional (linear) and landmark (geometric) morphometrics.

### 4.1 General shell morphology

During field work at Lake Lugu, the morphotypes which are considered *Margarya* and *Cipangopaludina* (Lu et al., 2014; Zhang et al., 2015) could be identified, however, they were interlinked by a wide variety of intermediate forms and thus the question arose whether the two genera may comprise several species or only a single, highly variable species, belonging to one monospecific genus represented by many phenotypes, present in Lake Lugu. The more intensive visual and the linear measurements (3.1) of 97 randomly selected shells from Lake Lugu confirm that either one highly variable species exists or that the shells can be rather subjectively assigned to three sculptural categories (Figure 4): weak = “*Cipangopaludina*,” strong = *“Margarya*,*”* and intermediate = *“Cipangopaludina*/*Margarya*.*”* The linear shell measurements of the Lake Dianchi *Margarya* are in the range of the Lake Lugu counterparts but this specimen plots rather distally, also well separated by landmark analysis. It can be speculated that measuring a couple of Lake Dianchi *Margarya* may result in a separate cluster. The type of sculpture of the Lake Dianchi *Margarya* specimen (Figure 2), however, is unique in the data set and distinct from the Lake Lugu taxa. The Lake Erhai *Cipangopaludina* (Figure 2) cannot be visually distinguished from the Lake Lugu *“Cipangopaludina*/*Margarya*.*”*

Embryonic shells of the Lake Lugu gastropods, which were studied from individuals of all sculptural categories, represent a single morphotype (Figure 4), which may support the idea of a single species.

### 4.2 Genetic analyses

Morphology‐based systematic assignments of living viviparids can be easily tested by genetic analyses. The study of fossil viviparids, which are, e.g., abundant and well preserved in Oligocene to Quaternary lacustrine sediments of southern and southwestern China (Tian et al., 2013; Yen, 1935; personal observations), has to focus on shell features though. The aim, however, is to bring genetic and shell data in line.

Our genetic results (3.4) show that *“Cipangopaludina*/*Margarya”* from Lake Lugu form a monophyletic clade which is possibly a lineage not closely related to other viviparid genera. Whether it is endemic to Lake Lugu has to remain open as, e.g., *Cipangopaludina* from Lake Erhai has not been genetically analyzed (see, e.g., Lu et al., 2014). Wiese et al. (2020) suggested an ongoing radiation of the gastropod genus *Radix* in Lake Lugu and found preliminary evidence that the gastropod genus *Gyraulus* may represent a species flock. Our data indicate that in the case of *“Cipangoplaudina*/*Margarya*,*”* only a single species is distributed over the lake. The multiple phenotypes cannot yet be distinguished genetically, at least not with mitochondrial markers. As a result, the possibility of an ongoing radiation for the larger viviparid species within Lake Lugu can neither be discarded nor proven.

### 4.3 Fractal dimension analyses

In contrast to the other shell morphological studies conducted here, fractal dimensions appear to be largely independent from visual reception.

The visual perception of size and sculpture is somewhat in agreement with the fractal dimensions considering the normal distribution of values but in disagreement when following the expectation that size and first‐order sculpture, which are commonly used in traditional morphometrics, should be reflected by fractal dimensions. As was shown, there is neither a correlation between max. size (height) and *D*_MB_ values nor can the strength of the first‐order sculpture (here, spiral keels) be clustered. The standard error, however, allows to separate the *Margarya* from Lake Dianchi, which on the other hand can be separated visually (Figure 2).

The fractal dimensions do not stand in contrast with the idea that a single, very variable *“Cipangopaludina*/*Margarya”* species exists in Lake Lugu as indicated by the genetic data and suggested by the traditional analysis of the morphotypes. The lack of correlation between *D*_MB_ values and visible shell morphologies led us to consider the second‐order sculpture. There is good evidence that in our technical setting (see methods) the number of spiral lirae correlates with *D*_MB_ values while first‐order sculpture plays a subordinate role. We suggest that the *D*_MB_ values are a measure of surface roughness. The definition of phenotypes by fractal dimensions and further aspects are discussed under 4.5.

### 4.4 Landmark analyses

We do not intend to reevaluate the performance of landmark analysis in gastropods, as Van Bocxlaer & Schultheiß (2010), but use this method to establish the fractal dimensions. At first glance, landmark results are in line with fractal dimensions: both data sets do not allow the separation of different Lake Lugu phenotypes (but Lake Dianchi *Margarya*) despite a trend from weakly to strongly sculptured forms (Figure 13a). Results of the fractal dimension and landmark analyses are difficult to compare, since our results suggest that both methods display different orders of morphological features. Landmarks represent main shell proportions and first‐order sculpture keels, whereas fractal dimensions seem to display second‐order sculpture, such as spiral lirae and growth increments. The landmark subset data of the northern and southern basins differ though (Figure 14), suggesting a basin‐dependent shift in morphospace occupation, which is not represented by fractal dimensions.

### 4.5 Advantages and limitations of fractal dimension analyses

The “power of 3D fractal dimensions” (Reichert et al., 2017) was demonstrated for corals which exhibit self‐similar branching structures of high complexity (Zawada et al., 2019). Reichert et al. (2017) emphasized that fractal dimensions performed better than “traditional methods” at the intra‐specific level. In non‐branching organisms such as ostracods, the valves of two species could be separated morphologically using fractal dimensions as well as with the aid of geometric measurements, but it was speculated that fractal dimensions can more appropriately capture micro‐sculpture (Aiello et al., 2007). These assumptions are in line with our observations. The fractal dimensions of the Lake Lugu gastropods appear to capture differences in second‐order sculpture, specifically the number of spiral lirae. The study, however, is not detailed enough to draw conclusions other than that the roughness of the shell surface is characterized mathematically. So far we can only speculate about the biological meaning of the amount of spiral lirae. It was observed in the field that algae were attached to all *“Cipangopaludina*/*Margarya”* shells but that density and type of algal growth were strongly varying. It is possible that shell surface roughness, expressed in the number of spiral lirae, controls algal attachment. We do not have such empirical data though because we did not systematically document the algal growth before the shells were cleaned. Future studies need to demonstrate the significance of our results. The next step will be then to relate these results with environmental parameters.

It has been emphasized by us (this study) and others (Aiello et al., 2007; Reichert et al., 2017) that one advantage of fractal dimensions lies in the primary independence from visual reception. This is only partly true and depends on the resolution of the 3D model. While the roughness of protein structures (Kaczor et al., 2012) is certainly beyond human perception, we are able to see the spiral lirae which the fractal dimensions captured. We just were ignorant about the meaning of second‐order sculpture. Fractal dimensions may open a new avenue of research which could lead to a higher level of understanding of gastropod ecology.

Regarding the question what value fractal dimensions add to describe shell phenotypes properly: It depends on the resolution of the 3D model (compare Reichert et al., 2017). In our setting, the quantification of micro‐sculpture (surface roughness) represents the major advantage over geometric analyses. We suppose that using a much lower resolution would lead to the “loss” of second‐order sculpture (spiral lirae) information in the data set and fractal dimensions would rather reflect the first‐order sculpture (spiral keels), which is surface roughness at a lower level. This hypothesis needs to be tested though. These considerations may also answer the question about the limitations of the method: It primarily describes the roughness of the shells' surfaces. To describe different levels, 3D models with different resolutions have to be produced which is quite time consuming.

We suggest that fractal dimension analyses using low‐resolution 3D models provide similar results as the geometric (here landmark) approach, while high‐resolution 3D models require a portfolio of methods including both, fractal dimension, and geometric analyses.

### 4.6 Implications for viviparid taxonomy in Lake Lugu

To date, from the group of larger viviparids, one *Angulyagra* species (Du et al., 2012), one *Margarya* species (Zhang et al., 2015), and one to two *Cipangopaludina* species (Wiese et al., 2020) are known from Lake Lugu. The two species *Angulyagra oxytropoides* and *Margarya oxytropoides* are synonyms (Zhang et al., 2015), which leaves a total number of two to three large viviparid species within the lake basins. Wiese et al. (2020) did not assign species names to the identified *Cipangopaludina* species and therefore, two valid viviparid genera are thought to be known from Lake Lugu. Strongly sculptured specimens from our study do clearly resemble *M. oxytropoides*, but however, this species assignment does not include the intermediate and weakly sculptured forms so far. Still, since the aim of this study was not to conduct a taxonomic revision of viviparid species from Lake Lugu, we suggest to refer to the specimens analyzed here as *Margarya oxytropoides*.

## 5 CONCLUSIONS

Although further studies are needed, we propose that fractal dimension analyses can be very useful to objectivize gastropod shell morphometrics in several respects. The major outcome is that (i) the values primarily describe the surface roughness of the shell. Hence, (ii) the resolution of the 3D model defines at which scale the surface roughness is calculated. A low‐resolution model may capture first‐order sculpture (but not second‐order sculpture) while a high‐resolution model (this study) captures second‐order sculpture (but not first‐order sculpture). (iii) A low‐resolution approach should resemble a geometric landmark analysis, with the advantage that subjective landmarking is avoided; (iv) a high‐resolution approach brings micro‐sculptures into focus (here spiral lirae). Since these are not captured by geometric morphometrics, this opens a new avenue for evolutionary and ecological considerations; (v) Shell preservation is important for the selection of 3D model resolutions; (vi) While an ongoing radiation can be observed in the basommatophoran genera *Radix* and *Gyraulus*, genetic analyses show that the morphologically diverse fauna of larger viviparids in Lake Lugu contains only one species.

## CONFLICT OF INTEREST

We have no conflict of interest to disclose.

## AUTHOR CONTRIBUTIONS

**Robert Wiese:** Conceptualization (equal); Data curation (lead); Investigation (equal); Methodology (equal); Validation (equal); Visualization (equal); Writing – original draft (equal). **Kyle Harrington:** Methodology (equal); Software (lead); Writing – review & editing (equal). **Kai Hartmann:** Conceptualization (equal); Data curation (equal); Investigation (equal); Methodology (equal); Visualization (equal); Writing – review & editing (equal). **Manja Hethke:** Conceptualization (equal); Formal analysis (equal); Investigation (equal); Methodology (equal); Visualization (equal); Writing – review & editing (equal). **Thomas von Rintelen:** Formal analysis (equal); Methodology (equal); Writing – review & editing (equal). **Hucai Zhang:** Formal analysis (equal); Methodology (equal); Writing – review & editing (equal). **Le‐Jia Zhang:** Formal analysis (equal); Methodology (equal); Writing – review & editing (equal). **Frank Riedel:** Conceptualization (equal); Formal analysis (equal); Investigation (equal); Methodology (equal); Supervision (equal); Writing – original draft (equal).

## Supporting information

Fig S1

Fig S2

Table S1

Table S2

Table S3

Table S4

Table S5

Data S1

Data S2

## Data Availability

All data used for the analyses are deposited in the Dryad data repository: Supplemental Data for Wiese et al., 2022, https://doi.org/10.5061/dryad.f1vhhmgxj.
