# Supplementary figures and images for: Can fractal dimensions objectivize gastropod shell morphometrics? A case study from Lake Lugu (SW China)
Source: Ecol Evol. 2022 Mar 1;12(3):e8622. doi: 10.1002/ece3.8622 (PMC8888252; doi:10.1002/ece3.8622)

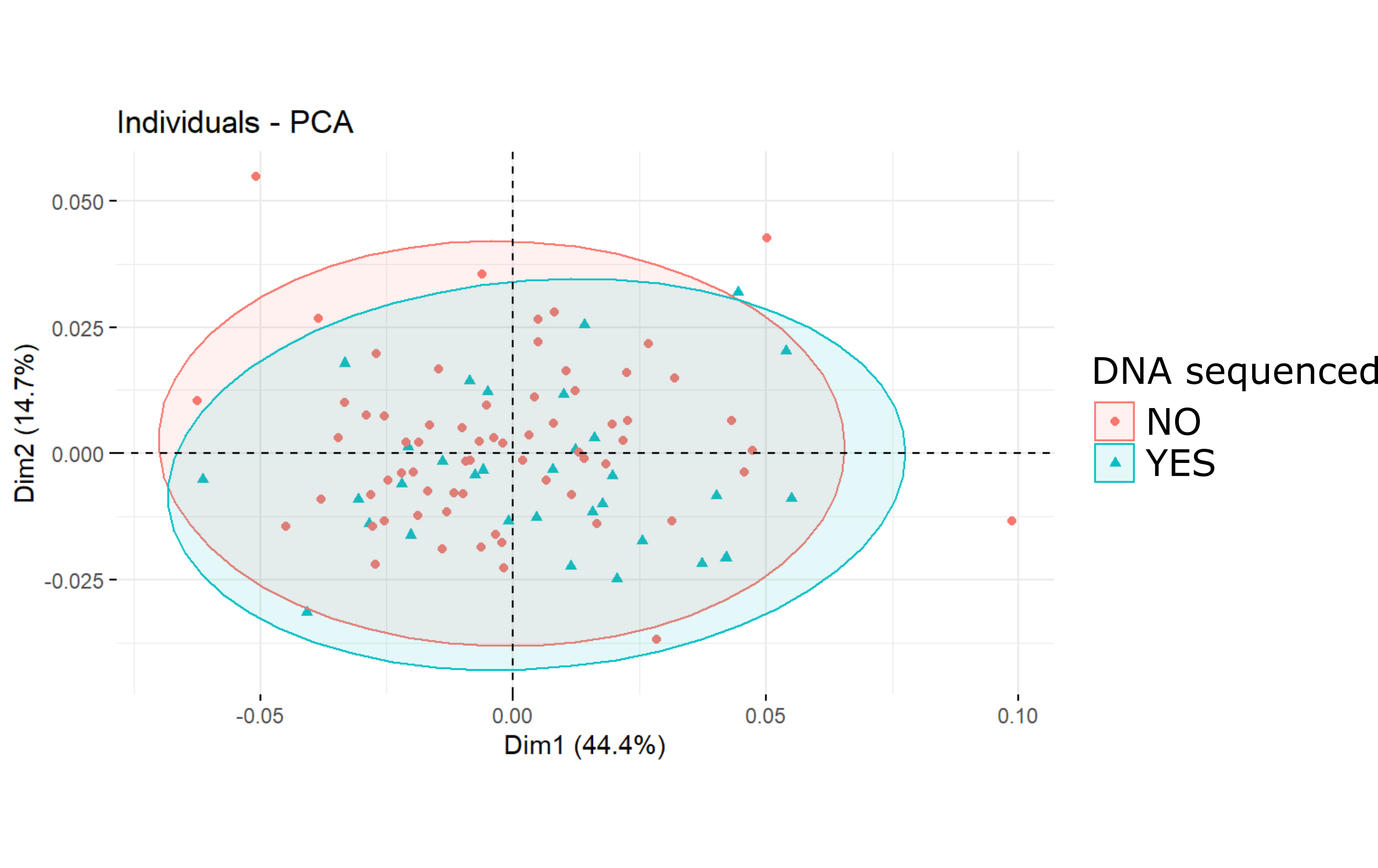

Supplement: Supplementary file 1 — Fig S1 [file ECE3-12-e8622-s003.png]

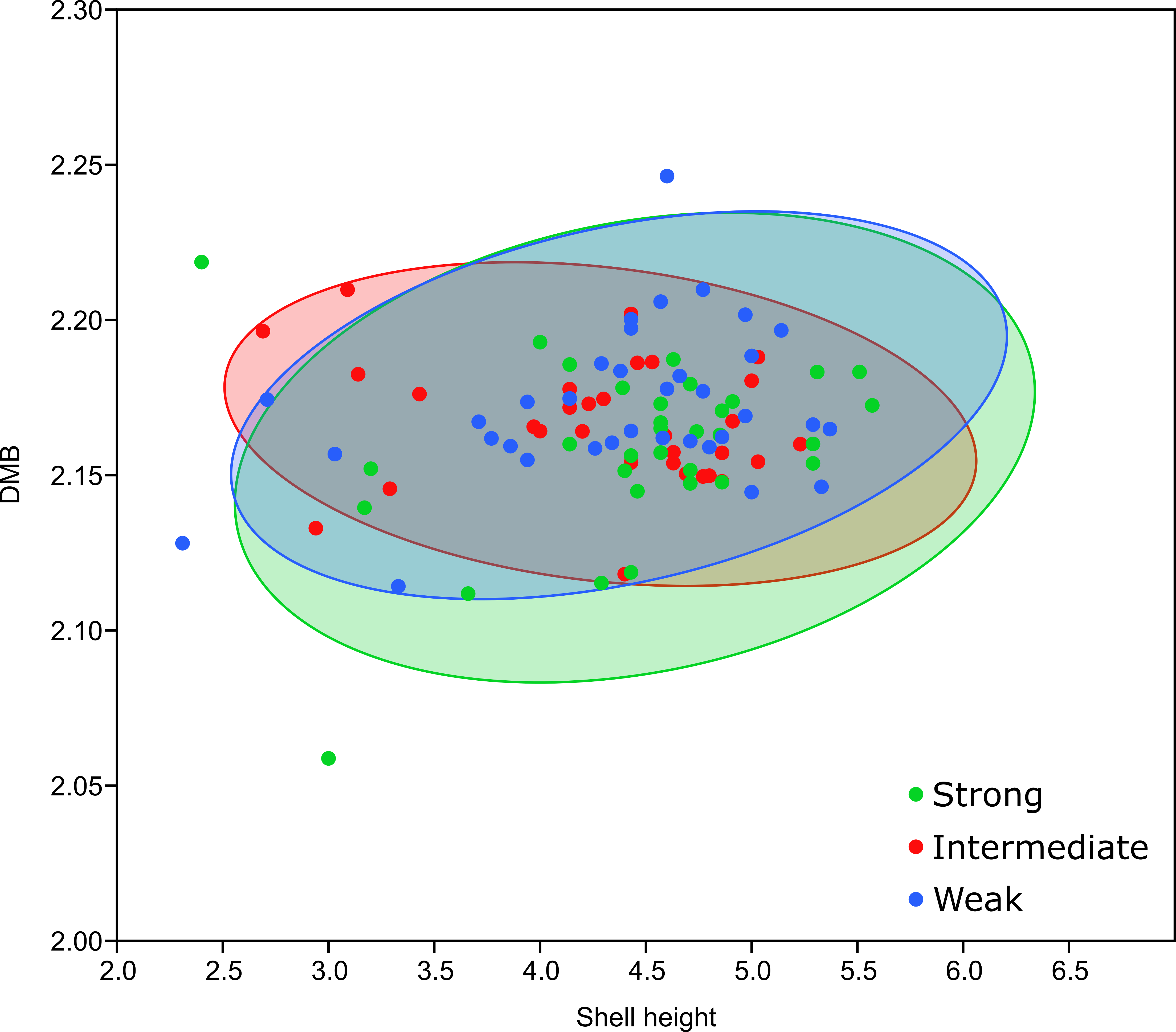

Supplement: Supplementary file 2 — Fig S2 [file ECE3-12-e8622-s009.png]
