# Supplementary material for: Can fractal dimensions objectivize gastropod shell morphometrics? A case study from Lake Lugu (SW China)
Source: Ecol Evol. 2022 Mar 1;12(3):e8622. doi: 10.1002/ece3.8622 (PMC8888252; doi:10.1002/ece3.8622)
Supplement: Supplementary file 8 — Data S1 [file ECE3-12-e8622-s007.html]

Lugu Landmark Analysis


Code 

- Show All Code
- Hide All Code

# Lugu Landmark Analysis

#### Manja Hethke, Kai Hartmann and Robert Wiese

#### 22.07.2021

**Most analyses based on package ‘geomorph’ (Adams et al., 2020), in parts following routine outlined by Theska et al. (2020).**

# 1 Read landmark data from tps file and definition of groupings

**99 individuals, 49 landmarks each**

```
##  [1] "1-113674_A"    "1-121321_2_1"  "1-121321_3"    "1-121322_2_2" 
##  [5] "1-121322_2_3"  "1-121322_2_4"  "1-121324_1"    "1-121328a_3"  
##  [9] "1-121328b_1"   "1-121328b_2_1" "1-121328b_2_4" "1-121328b_3"  
## [13] "1-121329a_2_1" "1-121329a_2_2" "1-121329b_1"   "1-121329b_3"  
## [17] "1-121330_2_1"  "1-121330_2_3"  "1-121330_A"    "1-121331_X"   
## [21] "1-121335_2_1"  "1-121335_B"    "1-121338_2_1"  "1-121338_A"   
## [25] "1-121340_1"    "1-121341_2_6"  "1-121342_2_3"  "1-121342_2_4" 
## [29] "1-121342_2_8"  "1-121344_A"    "1-121344_B"    "1-121344_C"   
## [33] "1-OUT_MARG"    "1-OUT_CIP"     "1-121321_1"    "1-121321_2_2" 
## [37] "1-121321_2"    "1-121322_2_1"  "1-121322_A"    "1-121322_B"   
## [41] "1-121322_C"    "1-121323_2_1"  "1-121323_2_2"  "1-121323_2_3" 
## [45] "1-121323_2_4"  "1-121323_2_5"  "1-121323_A"    "1-121323_B"   
## [49] "1-121328a_1"   "1-121328a_2"   "1-121328b_2_2" "1-121328b_2_3"
## [53] "1-121328b_2_5" "1-121328b_2_6" "1-121328b_2"   "1-121329a_1"  
## [57] "1-121329b_2"   "1-121330_2_2"  "1-121330_2_4"  "1-121330_B"   
## [61] "1-121331_A"    "1-121331_B"    "1-121335_2_2"  "1-121335_2_3" 
## [65] "1-121335_2_4"  "1-121335_2_5"  "1-121335_A"    "1-121335_C"   
## [69] "1-121337_1"    "1-121338_2_2"  "1-121338_2_3"  "1-121338_2_4" 
## [73] "1-121338_2_5"  "1-121340_2"    "1-121340_3"    "1-121340_4"   
## [77] "1-121341_2_1"  "1-121341_2_2"  "1-121341_2_3"  "1-121341_2_4" 
## [81] "1-121341_2_5"  "1-121341_2_7"  "1-121341_A"    "1-121342_1"   
## [85] "1-121342_2_1"  "1-121342_2_2"  "1-121342_2_5"  "1-121342_2_6" 
## [89] "1-121342_2_7"  "1-121342_2"    "1-121342_A"    "1-121344_2_1" 
## [93] "1-121438_A"    "1-127438_2_1"  "1-127438_2_2"  "1-127438_2_3" 
## [97] "1-127438_2_4"  "1-127438_2_5"  "1-127438_B"
```

```
## [1] 49  2 99
```

```
## [1] "Sculpture" "Sequenced"
```

```
##  [1] L S I I L S L I I L L I I S S S L L L S L L I S I I S L I L I I S L S S I I
## [39] S S S S I S S S S I L S L L L I I S S L L L S L L I L L L L L S I S I S I L
## [77] L I L L L I S I I I S L I S L I S I L I S L S
## Levels: I L S
```

**Sculpture groupings based on visual assessment.**

```
##  [1] YES YES YES YES YES YES YES YES YES YES YES YES YES YES YES YES YES YES YES
## [20] YES YES YES YES YES YES YES YES YES YES YES YES YES NO  NO  NO  NO  NO  NO 
## [39] NO  NO  NO  NO  NO  NO  NO  NO  NO  NO  NO  NO  NO  NO  NO  NO  NO  NO  NO 
## [58] NO  NO  NO  NO  NO  NO  NO  NO  NO  NO  NO  NO  NO  NO  NO  NO  NO  NO  NO 
## [77] NO  NO  NO  NO  NO  NO  NO  NO  NO  NO  NO  NO  NO  NO  NO  NO  NO  NO  NO 
## [96] NO  NO  NO  NO 
## Levels: NO YES
```

# 2 Data subsets

**Example: 34 2 99 = 34 landmarks, 2 dimensions (two coordinates per landmark), 99 gastropods**

```
## [1] 13  2 99
```

```
## [1] 10  2 99
```

```
## [1] 34  2 99
```

# 3 Analysis 1: Overall\_shape

## 3.1 Raw data plot

## 3.2 General Procrustes Analysis

**Performed on two-dimensional, fixed landmark coordinates using function gpagen {geomorph}**

**Procrustes superimposition: Shape independent of location, size and orientation.**

## 3.3 Landmark space

## 3.4 Outliers

### 3.4.1 tps grid deformation from mean shape (specimen 96 = 127438\_2\_3)

**Shape difference between the mean reference shape and the most extreme outliers and an example smooth form (OUT\_CIP)**

### 3.4.2 tps grid deformation from mean shape (specimen 92 = 1-121344\_2\_1)

### 3.4.3 tps grid deformation from mean shape (specimen 34 = 1-OUT\_CIP)

### 3.4.4 tps grid deformation from mean shape (specimen 33 = 1-OUT\_MARG)

## 3.5 Shape space

```
## Importance of components:
##                            PC1     PC2     PC3     PC4      PC5      PC6
## Standard deviation     0.02757 0.01589 0.01324 0.01063 0.008798 0.007764
## Proportion of Variance 0.44382 0.14738 0.10228 0.06600 0.045190 0.035200
## Cumulative Proportion  0.44382 0.59120 0.69348 0.75948 0.804670 0.839860
##                             PC7      PC8      PC9     PC10     PC11     PC12
## Standard deviation     0.007353 0.006607 0.006207 0.005415 0.004972 0.004584
## Proportion of Variance 0.031570 0.025480 0.022490 0.017120 0.014430 0.012270
## Cumulative Proportion  0.871430 0.896910 0.919410 0.936520 0.950950 0.963220
##                            PC13     PC14     PC15     PC16     PC17     PC18
## Standard deviation     0.003968 0.003444 0.002934 0.002555 0.002375 0.002035
## Proportion of Variance 0.009190 0.006930 0.005030 0.003810 0.003290 0.002420
## Cumulative Proportion  0.972410 0.979340 0.984370 0.988180 0.991470 0.993890
##                            PC19     PC20     PC21     PC22      PC23      PC24
## Standard deviation     0.001901 0.001764 0.001482 0.001242 1.977e-16 1.389e-16
## Proportion of Variance 0.002110 0.001820 0.001280 0.000900 0.000e+00 0.000e+00
## Cumulative Proportion  0.996000 0.997820 0.999100 1.000000 1.000e+00 1.000e+00
##                             PC25      PC26
## Standard deviation     8.612e-17 3.261e-17
## Proportion of Variance 0.000e+00 0.000e+00
## Cumulative Proportion  1.000e+00 1.000e+00
```

### 3.5.1 Barplot indicating the variation described by principal components

**red line = mean variation**

### 3.5.2 Identification of number of meaningful principal components

```
##  [1] 4.438183e+01 1.473786e+01 1.022835e+01 6.600023e+00 4.518810e+00
##  [6] 3.519593e+00 3.156649e+00 2.548275e+00 2.249285e+00 1.711715e+00
## [11] 1.443021e+00 1.226959e+00 9.190360e-01 6.925796e-01 5.027501e-01
## [16] 3.810783e-01 3.292884e-01 2.418634e-01 2.110565e-01 1.815711e-01
## [21] 1.283034e-01 9.009796e-02 2.280912e-27 1.125983e-27 4.329813e-28
## [26] 6.207927e-29
```

```
## $tol
## [1] 1.329425
## 
## $good
## [1] 1 2 3 4
```

**number of meaningful principal components = 4**

### 3.5.3 PCA of shape variables

**Sculpture groups L, I, S**

**DNA sequenced yes/no**

### 3.5.4 Lollipop plot of superimposed extreme shapes on PC1 and PC2

### 3.5.5 Contribution of landmarks on principal components (%)

```
PC2_contrib <- fviz_contrib(lugu.pca, choice = "var", axes = 2)
LM_contrib_PC2 <- data.frame(LM = rep(NA, nrow(PC2_contrib$data)/2), PC2_contrib = rep(NA, nrow(PC2_contrib$data)/2))
for (i in 1:(nrow(PC2_contrib$data)/2)) {  
  LM_contrib_PC2$PC2_contrib[i] <- sum(PC2_contrib$data[(i*2-1):(i*2),]$contrib)
  LM_contrib_PC2$LM[i] <- as.character(i)
}
important_LMs_PC1 <- LM_contrib_PC1[LM_contrib_PC1$PC1_contrib > 100/(nrow(PC1_contrib$data)/2),]
important_LMs_PC1[order(decreasing = TRUE, important_LMs_PC1$PC1_contrib),]
```

```
##    LM PC1_contrib
## 9   9   18.171720
## 12 12   16.313943
## 10 10   12.163689
## 11 11   11.770819
## 5   5    7.845882
```

```
important_LMs_PC2 <- LM_contrib_PC2[LM_contrib_PC2$PC2_contrib > 100/(nrow(PC2_contrib$data)/2),]
important_LMs_PC2[order(decreasing = TRUE, important_LMs_PC2$PC2_contrib),]
```

```
##    LM PC2_contrib
## 12 12    64.67223
## 10 10    12.68280
```

# 4 Analysis 2: Sculpture\_reduced

## 4.1 Raw data plot

## 4.2 General Procrustes Analysis

**Performed on two-dimensional, fixed landmark coordinates using function gpagen {geomorph}**

**Procrustes superimposition: Shape independent of location, size and orientation.**

## 4.3 Landmark space

## 4.4 Outliers

```
##    1-OUT_MARG  1-127438_2_3  1-121321_2_1   1-121328b_1   1-121328a_1 
##            33            96             2             9            49 
##    1-121438_A  1-121322_2_3    1-121340_4  1-121323_2_2 1-121328b_2_5 
##            93             5            76            43            53 
##  1-121335_2_2 1-121328b_2_2    1-121331_B    1-121335_C  1-127438_2_2 
##            63            51            62            68            95 
##  1-121330_2_3    1-121331_X  1-121322_2_2  1-121335_2_5  1-121323_2_3 
##            18            20             4            66            44 
##    1-121321_3   1-121329a_1  1-121342_2_6  1-121323_2_1  1-121342_2_3 
##             3            56            88            42            27 
##  1-127438_2_4    1-121342_A 1-121329a_2_1  1-121341_2_7    1-121321_1 
##            97            91            13            82            35 
##  1-121323_2_5  1-121341_2_4    1-121341_A 1-121328b_2_6    1-121330_A 
##            46            80            83            54            19 
##    1-121330_B   1-121329b_3    1-121335_A    1-121324_1    1-121335_B 
##            60            16            67             7            22 
##  1-121338_2_5 1-121328b_2_3  1-121342_2_7    1-121323_A  1-121338_2_1 
##            73            52            89            47            23 
##  1-121342_2_1   1-121328a_2  1-121335_2_1  1-121341_2_6  1-127438_2_5 
##            85            50            21            26            98 
##  1-121321_2_2     1-OUT_CIP  1-121341_2_1  1-121342_2_8 1-121328b_2_4 
##            36            34            77            29            11 
##    1-121321_2  1-121330_2_4  1-127438_2_1  1-121342_2_5   1-121328a_3 
##            37            59            94            87             8 
##    1-127438_B    1-113674_A    1-121322_C    1-121344_C  1-121341_2_2 
##            99             1            41            32            78 
##  1-121338_2_3  1-121323_2_4    1-121322_A    1-121344_A    1-121340_3 
##            71            45            39            30            75 
##  1-121335_2_3    1-121337_1  1-121322_2_4   1-121329b_2    1-121340_2 
##            64            69             6            57            74 
##    1-121340_1  1-121335_2_4    1-121342_1   1-121328b_2  1-121341_2_3 
##            25            65            84            55            79 
##   1-121329b_1  1-121330_2_1  1-121330_2_2    1-121323_B  1-121342_2_2 
##            15            17            58            48            86 
##    1-121342_2  1-121344_2_1  1-121338_2_4    1-121322_B  1-121322_2_1 
##            90            92            72            40            38 
##  1-121341_2_5 1-121328b_2_1    1-121331_A    1-121344_B  1-121342_2_4 
##            81            10            61            31            28 
##   1-121328b_3  1-121338_2_2    1-121338_A 1-121329a_2_2 
##            12            70            24            14
```

### 4.4.1 tps grid deformation from mean shape (specimen 96 = 127438\_2\_3)

**Shape difference between the mean reference shape and the most extreme outliers and an example smooth form (OUT\_CIP)**

### 4.4.2 tps grid deformation from mean shape (specimen 92 = 1-121344\_2\_1)

### 4.4.3 tps grid deformation from mean shape (specimen 34 = 1-OUT\_CIP)

### 4.4.4 tps grid deformation from mean shape (specimen 33 = 1-OUT\_MARG)

### 4.4.5 tps grid deformation from mean shape (specimen 2 = 1-121321\_2\_1, most neg score on PC1)

### 4.4.6 tps grid deformation from mean shape (specimen 5 = 1-121322\_2\_3, 2nd most neg score on PC2)

### 4.4.7 tps grid deformation from mean shape (specimen 76 = 1-121340\_4, most pos score on PC2)

## 4.5 Shape space

```
## Importance of components:
##                            PC1     PC2     PC3     PC4      PC5      PC6
## Standard deviation     0.02777 0.01903 0.01551 0.01132 0.009336 0.008187
## Proportion of Variance 0.41788 0.19622 0.13040 0.06942 0.047230 0.036320
## Cumulative Proportion  0.41788 0.61410 0.74450 0.81392 0.861160 0.897480
##                             PC7      PC8      PC9     PC10     PC11     PC12
## Standard deviation     0.006958 0.005646 0.005349 0.004835 0.003649 0.003618
## Proportion of Variance 0.026230 0.017280 0.015500 0.012670 0.007220 0.007090
## Cumulative Proportion  0.923720 0.940990 0.956500 0.969170 0.976380 0.983470
##                            PC13     PC14     PC15     PC16      PC17      PC18
## Standard deviation     0.003396 0.002903 0.002433 0.002149 1.785e-16 1.131e-16
## Proportion of Variance 0.006250 0.004570 0.003210 0.002500 0.000e+00 0.000e+00
## Cumulative Proportion  0.989720 0.994290 0.997500 1.000000 1.000e+00 1.000e+00
##                            PC19     PC20
## Standard deviation     7.32e-17 3.28e-17
## Proportion of Variance 0.00e+00 0.00e+00
## Cumulative Proportion  1.00e+00 1.00e+00
```

### 4.5.1 Barplot indicating the variation described by principal components

**red line = mean variation**

### 4.5.2 Identification of number of meaningful principal components

```
##  [1] 4.178762e+01 1.962207e+01 1.304049e+01 6.942267e+00 4.723403e+00
##  [6] 3.632295e+00 2.623479e+00 1.727562e+00 1.550380e+00 1.266946e+00
## [11] 7.216820e-01 7.092099e-01 6.249361e-01 4.567144e-01 3.206373e-01
## [16] 2.503160e-01 1.726056e-27 6.933844e-28 2.903404e-28 5.828669e-29
```

```
## $tol
## [1] 1.329425
## 
## $good
## [1] 1 2 3 4
```

**number of meaningful principal components = 4**

### 4.5.3 PCA of shape variables

**Sculpture groups L, I, S**

**DNA sequenced yes/no**

**Little discrimination between the three visual sculpture groups I, L and S. That’s quite unexpected.**

### 4.5.4 Lollipop plot of superimposed extreme shapes on PC1 and PC2

### 4.5.5 Contribution of landmarks on principal components (%)

```
##   LM PC1_contrib
## 6  6    19.35918
## 7  7    13.80565
## 1  1    11.59666
## 2  2    10.00430
```

```
##    LM PC2_contrib
## 7   7    24.60152
## 6   6    22.80727
## 5   5    15.99569
## 4   4    14.34012
## 10 10    10.76605
```

# 5 Analysis 3: Full\_set

## 5.1 Raw data plot

## 5.2 General Procrustes Analysis

**Performed on two-dimensional, fixed landmark coordinates using function gpagen {geomorph}**

**Procrustes superimposition: Shape independent of location, size and orientation.**

## 5.3 Landmark space

## 5.4 Outliers

```
##    1-OUT_MARG  1-127438_2_3  1-121321_2_1  1-121338_2_5    1-121335_C 
##            33            96             2            73            68 
##  1-121322_2_2    1-121324_1    1-121438_A    1-121331_B  1-127438_2_4 
##             4             7            93            62            97 
##   1-121328a_1    1-121340_4 1-121329a_2_1  1-121344_2_1 1-121328b_2_5 
##            49            76            13            92            53 
##    1-121335_B  1-121322_2_3  1-121335_2_5  1-121323_2_3    1-121331_X 
##            22             5            66            44            20 
##  1-127438_2_2  1-121323_2_2    1-121342_A  1-121323_2_5  1-121338_2_1 
##            95            43            91            46            23 
##  1-121342_2_6  1-121341_2_4  1-121342_2_8   1-121328b_1  1-121330_2_1 
##            88            80            29             9            17 
##     1-OUT_CIP    1-121321_2    1-121341_A  1-121341_2_6    1-121321_3 
##            34            37            83            26             3 
##   1-121329a_1    1-121321_1    1-121323_A   1-121328a_3  1-127438_2_1 
##            56            35            47             8            94 
##  1-121335_2_2  1-121342_2_1  1-121323_2_1 1-121328b_2_2  1-121341_2_1 
##            63            85            42            51            77 
##    1-121344_C    1-121330_B    1-121323_B    1-121340_1 1-121328b_2_6 
##            32            60            48            25            54 
##    1-113674_A  1-127438_2_5  1-121330_2_3  1-121342_2_7  1-121335_2_1 
##             1            98            18            89            21 
##  1-121338_2_2    1-121340_3  1-121321_2_2    1-127438_B  1-121338_2_3 
##            70            75            36            99            71 
##  1-121342_2_2   1-121328a_2    1-121330_A  1-121341_2_2  1-121330_2_4 
##            86            50            19            78            59 
## 1-121328b_2_3    1-121344_A   1-121329b_1  1-121335_2_3  1-121341_2_7 
##            52            30            15            64            82 
##  1-121323_2_4 1-121328b_2_4  1-121322_2_4    1-121342_1    1-121335_A 
##            45            11             6            84            67 
##  1-121342_2_3   1-121329b_2  1-121330_2_2  1-121341_2_5  1-121342_2_5 
##            27            57            58            81            87 
##    1-121338_A   1-121328b_3   1-121329b_3  1-121335_2_4    1-121340_2 
##            24            12            16            65            74 
##   1-121328b_2 1-121329a_2_2  1-121322_2_1    1-121322_C  1-121341_2_3 
##            55            14            38            41            79 
## 1-121328b_2_1  1-121338_2_4  1-121342_2_4    1-121337_1    1-121342_2 
##            10            72            28            69            90 
##    1-121322_A    1-121344_B    1-121331_A    1-121322_B 
##            39            31            61            40
```

### 5.4.1 tps grid deformation from mean shape (specimen 96 = 127438\_2\_3)

**Shape difference between the mean reference shape and the most extreme outliers and an example smooth form (OUT\_CIP)**

### 5.4.2 tps grid deformation from mean shape (specimen 92 = 1-121344\_2\_1)

### 5.4.3 tps grid deformation from mean shape (specimen 34 = 1-OUT\_CIP)

### 5.4.4 tps grid deformation from mean shape (specimen 33 = 1-OUT\_MARG)

## 5.5 Shape space

```
## Importance of components:
##                            PC1     PC2     PC3     PC4     PC5     PC6      PC7
## Standard deviation     0.03146 0.01908 0.01526 0.01268 0.01122 0.01102 0.009741
## Proportion of Variance 0.35669 0.13124 0.08391 0.05795 0.04536 0.04375 0.034200
## Cumulative Proportion  0.35669 0.48793 0.57185 0.62980 0.67516 0.71891 0.753120
##                             PC8      PC9     PC10     PC11     PC12     PC13
## Standard deviation     0.008184 0.007989 0.007365 0.007096 0.006638 0.006455
## Proportion of Variance 0.024140 0.023000 0.019550 0.018150 0.015880 0.015020
## Cumulative Proportion  0.777250 0.800260 0.819810 0.837960 0.853840 0.868860
##                            PC14     PC15    PC16     PC17     PC18     PC19
## Standard deviation     0.005689 0.005575 0.00541 0.005161 0.004991 0.004506
## Proportion of Variance 0.011660 0.011200 0.01055 0.009600 0.008980 0.007320
## Cumulative Proportion  0.880520 0.891720 0.90227 0.911870 0.920850 0.928170
##                            PC20     PC21     PC22     PC23     PC24     PC25
## Standard deviation     0.004299 0.004119 0.003886 0.003694 0.003528 0.003448
## Proportion of Variance 0.006660 0.006110 0.005440 0.004920 0.004490 0.004290
## Cumulative Proportion  0.934830 0.940950 0.946390 0.951310 0.955790 0.960080
##                            PC26     PC27     PC28     PC29    PC30     PC31
## Standard deviation     0.003143 0.003061 0.003006 0.002768 0.00270 0.002519
## Proportion of Variance 0.003560 0.003380 0.003260 0.002760 0.00263 0.002290
## Cumulative Proportion  0.963640 0.967020 0.970270 0.973040 0.97566 0.977950
##                            PC32     PC33     PC34     PC35     PC36     PC37
## Standard deviation     0.002407 0.002324 0.002248 0.002055 0.002005 0.001864
## Proportion of Variance 0.002090 0.001950 0.001820 0.001520 0.001450 0.001250
## Cumulative Proportion  0.980040 0.981990 0.983810 0.985330 0.986780 0.988030
##                            PC38     PC39     PC40     PC41     PC42     PC43
## Standard deviation     0.001767 0.001658 0.001641 0.001581 0.001503 0.001423
## Proportion of Variance 0.001130 0.000990 0.000970 0.000900 0.000810 0.000730
## Cumulative Proportion  0.989160 0.990150 0.991120 0.992020 0.992830 0.993560
##                            PC44     PC45     PC46     PC47     PC48     PC49
## Standard deviation     0.001406 0.001329 0.001246 0.001209 0.001198 0.001147
## Proportion of Variance 0.000710 0.000640 0.000560 0.000530 0.000520 0.000470
## Cumulative Proportion  0.994280 0.994910 0.995470 0.996000 0.996520 0.996990
##                           PC50      PC51      PC52      PC53      PC54
## Standard deviation     0.00103 0.0009925 0.0009659 0.0009183 0.0008343
## Proportion of Variance 0.00038 0.0003600 0.0003400 0.0003000 0.0002500
## Cumulative Proportion  0.99737 0.9977300 0.9980600 0.9983700 0.9986200
##                             PC55      PC56      PC57      PC58      PC59
## Standard deviation     0.0008193 0.0007794 0.0007639 0.0006452 0.0006328
## Proportion of Variance 0.0002400 0.0002200 0.0002100 0.0001500 0.0001400
## Cumulative Proportion  0.9988600 0.9990800 0.9992900 0.9994400 0.9995800
##                             PC60     PC61      PC62      PC63      PC64
## Standard deviation     0.0005829 0.000556 0.0004514 0.0004149 0.0003571
## Proportion of Variance 0.0001200 0.000110 0.0000700 0.0000600 0.0000500
## Cumulative Proportion  0.9997100 0.999820 0.9998900 0.9999500 1.0000000
##                             PC65      PC66      PC67      PC68
## Standard deviation     1.541e-16 4.346e-17 3.523e-17 2.068e-17
## Proportion of Variance 0.000e+00 0.000e+00 0.000e+00 0.000e+00
## Cumulative Proportion  1.000e+00 1.000e+00 1.000e+00 1.000e+00
```

### 5.5.1 Barplot indicating the variation described by principal components

**red line = mean variation**

### 5.5.2 Identification of number of meaningful principal components

```
##  [1] 3.566905e+01 1.312443e+01 8.391252e+00 5.795419e+00 4.535574e+00
##  [6] 4.375473e+00 3.420405e+00 2.413865e+00 2.300362e+00 1.955160e+00
## [11] 1.814771e+00 1.588133e+00 1.501820e+00 1.166470e+00 1.120225e+00
## [16] 1.054878e+00 9.600532e-01 8.978311e-01 7.319216e-01 6.660304e-01
## [21] 6.114580e-01 5.444360e-01 4.918185e-01 4.485957e-01 4.285894e-01
## [26] 3.560819e-01 3.377277e-01 3.255998e-01 2.761840e-01 2.628573e-01
## [31] 2.288025e-01 2.088432e-01 1.946256e-01 1.821565e-01 1.522110e-01
## [36] 1.448982e-01 1.252212e-01 1.125256e-01 9.912279e-02 9.701187e-02
## [41] 9.007761e-02 8.138107e-02 7.301551e-02 7.128279e-02 6.363287e-02
## [46] 5.597993e-02 5.264923e-02 5.177088e-02 4.737946e-02 3.823936e-02
## [51] 3.550263e-02 3.362794e-02 3.039344e-02 2.508575e-02 2.419260e-02
## [56] 2.189478e-02 2.103118e-02 1.500493e-02 1.443536e-02 1.224522e-02
## [61] 1.114299e-02 7.345632e-03 6.203921e-03 4.597095e-03 8.558347e-28
## [66] 6.809021e-29 4.473211e-29 1.541363e-29
```

```
## $tol
## [1] 1.329425
## 
## $good
## [1] 1 2 3
```

**number of meaningful principal components = 3**

### 5.5.3 PCA of shape variables

**Sculpture groups L, I, S**

**DNA sequenced yes/no**

### 5.5.4 Lollipop plot of superimposed extreme shapes on PC1 and PC2

### 5.5.5 Contribution of landmarks on principal components (%)

```
##    LM PC1_contrib
## 16 16    9.988980
## 15 15    8.599067
## 17 17    8.411992
## 14 14    6.903180
## 32 32    6.593102
## 18 18    5.490817
## 30 30    5.249099
## 28 28    4.449743
## 27 27    4.145614
## 29 29    4.075704
## 26 26    4.024430
## 2   2    3.975830
## 1   1    3.949948
## 31 31    3.321723
## 4   4    3.298029
## 3   3    3.000399
```

```
##    LM PC2_contrib
## 26 26   15.010466
## 21 21   14.128936
## 32 32    9.973466
## 23 23    7.547510
## 31 31    6.871779
## 22 22    6.816225
## 6   6    6.620812
## 11 11    5.522807
## 25 25    3.535296
```
