# Supplementary material for: Can fractal dimensions objectivize gastropod shell morphometrics? A case study from Lake Lugu (SW China)
Source: Ecol Evol. 2022 Mar 1;12(3):e8622. doi: 10.1002/ece3.8622 (PMC8888252; doi:10.1002/ece3.8622)
Supplement: Supplementary file 9 — Data S2 [file ECE3-12-e8622-s001.html]

Lugu Landmark Analysis: North and South Basin


Code 

- Show All Code
- Hide All Code

# Lugu Landmark Analysis: North and South Basin

#### Manja Hethke, Kai Hartmann and Robert Wiese

#### 22.07.2021

**Most analyses based on package ‘geomorph’ (Adams et al., 2020), in parts following routine outlined by Theska et al. (2020).**

# 1 Read landmark data from tps file and definition of groupings

**97 individuals, 49 landmarks each (individuals of Lake Lugu, only)**

```
##  [1] "N-113674_A-1-2"    "S-121321_2_1-1-2"  "S-121321_3-1-2"   
##  [4] "N-121322_2_2-1-2"  "N-121322_2_3-1-2"  "N-121322_2_4-1-2" 
##  [7] "N-121324_1-1-2"    "N-121328a_3-1-2"   "N-121328b_1-1-2"  
## [10] "N-121328b_2_1-1-2" "N-121328b_2_4-1-2" "N-121328b_3-1-2"  
## [13] "N-121329a_2_1-1-2" "N-121329a_2_2-1-2" "N-121329b_1-1-2"  
## [16] "N-121329b_3-1-2"   "N-121330_2_1-1-2"  "N-121330_2_3-1-2" 
## [19] "N-121330_A-1-2"    "S-121331_X-1-2"    "N-121335_2_1-1-2" 
## [22] "N-121335_B-1-2"    "S-121338_2_1-1-2"  "S-121338_A-1-2"   
## [25] "S-121340_1-1-2"    "N-121341_2_6-1-2"  "N-121342_2_3-1-2" 
## [28] "N-121342_2_4-1-2"  "N-121342_2_8-1-2"  "N-121344_A-1-2"   
## [31] "N-121344_B-1-2"    "N-121344_C-1-2"    "S-121321_1-1-2"   
## [34] "S-121321_2_2-1-2"  "S-121321_2-1-2"    "N-121322_2_1-1-2" 
## [37] "N-121322_A-1-2"    "N-121322_B-1-2"    "N-121322_C-1-2"   
## [40] "S-121323_2_1-1-2"  "S-121323_2_2-1-2"  "S-121323_2_3-1-2" 
## [43] "S-121323_2_4-1-2"  "S-121323_2_5-1-2"  "S-121323_A-1-2"   
## [46] "S-121323_B-1-2"    "N-121328a_1-1-2"   "N-121328a_2-1-2"  
## [49] "N-121328b_2_2-1-2" "N-121328b_2_3-1-2" "N-121328b_2_5-1-2"
## [52] "N-121328b_2_6-1-2" "N-121328b_2-1-2"   "N-121329a_1-1-2"  
## [55] "N-121329b_2-1-2"   "N-121330_2_2-1-2"  "N-121330_2_4-1-2" 
## [58] "N-121330_B-1-2"    "S-121331_A-1-2"    "S-121331_B-1-2"   
## [61] "N-121335_2_2-1-2"  "N-121335_2_3-1-2"  "N-121335_2_4-1-2" 
## [64] "N-121335_2_5-1-2"  "N-121335_A-1-2"    "N-121335_C-1-2"   
## [67] "N-121337_1-1-2"    "S-121338_2_2-1-2"  "S-121338_2_3-1-2" 
## [70] "S-121338_2_4-1-2"  "S-121338_2_5-1-2"  "S-121340_2-1-2"   
## [73] "S-121340_3-1-2"    "S-121340_4-1-2"    "N-121341_2_1-1-2" 
## [76] "N-121341_2_2-1-2"  "N-121341_2_3-1-2"  "N-121341_2_4-1-2" 
## [79] "N-121341_2_5-1-2"  "N-121341_2_7-1-2"  "N-121341_A-1-2"   
## [82] "N-121342_1-1-2"    "N-121342_2_1-1-2"  "N-121342_2_2-1-2" 
## [85] "N-121342_2_5-1-2"  "N-121342_2_6-1-2"  "N-121342_2_7-1-2" 
## [88] "N-121342_2-1-2"    "N-121342_A-1-2"    "N-121344_2_1-1-2" 
## [91] "S-121438_A-1-2"    "S-127438_2_1-1-2"  "S-127438_2_2-1-2" 
## [94] "S-127438_2_3-1-2"  "S-127438_2_4-1-2"  "S-127438_2_5-1-2" 
## [97] "S-127438_B-1-2"
```

```
## [1] 49  2 97
```

```
## [1] "Sculpture" "Sequenced"
```

```
##  [1] L S I I L S L I I L L I I S S S L L L S L L I S I I S L I L I I S S I I S S
## [39] S S I S S S S I L S L L L I I S S L L L S L L I L L L L L S I S I S I L L I
## [77] L L L I S I I I S L I S L I S I L I S L S
## Levels: I L S
```

**Sculpture groupings based on visual assessment.**

```
##  [1] YES YES YES YES YES YES YES YES YES YES YES YES YES YES YES YES YES YES YES
## [20] YES YES YES YES YES YES YES YES YES YES YES YES YES NO  NO  NO  NO  NO  NO 
## [39] NO  NO  NO  NO  NO  NO  NO  NO  NO  NO  NO  NO  NO  NO  NO  NO  NO  NO  NO 
## [58] NO  NO  NO  NO  NO  NO  NO  NO  NO  NO  NO  NO  NO  NO  NO  NO  NO  NO  NO 
## [77] NO  NO  NO  NO  NO  NO  NO  NO  NO  NO  NO  NO  NO  NO  NO  NO  NO  NO  NO 
## [96] NO  NO 
## Levels: NO YES
```

# 2 Data subsets

**Example: 13 2 97 = 13 landmarks, 2 dimensions (two coordinates per landmark), 97 gastropods**

```
## [1] 13  2 97
```

```
## [1] 10  2 97
```

```
## [1] 34  2 97
```

# 3 Analysis 1: Full\_set

## 3.1 Raw data plot

## 3.2 General Procrustes Analysis

**Performed on two-dimensional, fixed landmark coordinates using function gpagen {geomorph}**

**Procrustes superimposition: Shape independent of location, size and orientation.**

## 3.3 Landmark space

**N will be red and S will be blue in the landmark-space plot below**

## 3.4 Statistical tests

**Procrustes ANOVA (performed by function procD.lm) based on Euclidean distances from the generalized procrustes analysis (gpa) above**

```
## 
## Analysis of Variance, using Residual Randomization
## Permutation procedure: Randomization of raw values (residuals of mean) 
## Number of permutations: 100001 
## Estimation method: Ordinary Least Squares 
## Sums of Squares and Cross-products: Type I 
## Effect sizes (Z) based on F distributions
## 
##           Df       SS        MS     Rsq      F      Z Pr(>F)    
## locality   1 0.026335 0.0263346 0.10289 10.896 5.1655  1e-05 ***
## Residuals 95 0.229604 0.0024169 0.89711                         
## Total     96 0.255938                                           
## ---
## Signif. codes:  0 '***' 0.001 '**' 0.01 '*' 0.05 '.' 0.1 ' ' 1
## 
## Call: procD.lm(f1 = shape ~ locality, iter = 1e+05, seed = 12345, RRPP = F,  
##     effect.type = "F", data = gdf)
```

### 3.4.1 Pairwise Procrustes Anova

```
## 
## Pairwise comparisons
## 
## Groups: N S 
## 
## FRPP: 100001 permutations
## 
## LS means:
## Vectors hidden (use show.vectors = TRUE to view)
## 
## Pairwise distances between means, plus statistics
##              d  UCL (95%)        Z     Pr > d
## N:S 0.03504429 0.01602353 8.854909 9.9999e-06
```

```
##            N          S
## N 1.0000e+00 9.9999e-06
## S 9.9999e-06 1.0000e+00
```

### 3.4.2 False discovery rate (FDR) using p.adjust function: Correction of p-values obtained from pairwise Procrustes Anova

```
##   N (FDR-adj.) S (FDR-adj.)
## N  1.00000e+00  1.99998e-05
## S  1.99998e-05  1.00000e+00
```

## 3.5 Outliers

**Procrustes distances of specimens to mean shape, outliers in red**

```
##  S-127438_2_3-1-2  S-121321_2_1-1-2  S-121338_2_5-1-2    N-121335_C-1-2 
##                94                 2                71                66 
##  N-121322_2_2-1-2    N-121324_1-1-2    S-121438_A-1-2    S-121331_B-1-2 
##                 4                 7                91                60 
##  S-127438_2_4-1-2   N-121328a_1-1-2    S-121340_4-1-2 N-121328b_2_5-1-2 
##                95                47                74                51 
## N-121329a_2_1-1-2  N-121344_2_1-1-2    N-121335_B-1-2  N-121322_2_3-1-2 
##                13                90                22                 5 
##  N-121335_2_5-1-2  S-121323_2_3-1-2  S-121323_2_2-1-2    S-121331_X-1-2 
##                64                42                41                20 
##    N-121342_A-1-2  S-121323_2_5-1-2  S-127438_2_2-1-2  S-121338_2_1-1-2 
##                89                44                93                23 
##  N-121342_2_6-1-2  N-121342_2_8-1-2  N-121341_2_4-1-2  N-121330_2_1-1-2 
##                86                29                78                17 
##   N-121328b_1-1-2    S-121321_2-1-2   N-121329a_1-1-2    N-121341_A-1-2 
##                 9                35                54                81 
##  N-121341_2_6-1-2    S-121321_1-1-2  N-121335_2_2-1-2    S-121321_3-1-2 
##                26                33                61                 3 
##    S-121323_A-1-2  N-121342_2_1-1-2   N-121328a_3-1-2 N-121328b_2_2-1-2 
##                45                83                 8                49 
##  S-127438_2_1-1-2  N-121341_2_1-1-2  S-121323_2_1-1-2    N-121344_C-1-2 
##                92                75                40                32 
##    N-121330_B-1-2    S-121323_B-1-2    S-121340_1-1-2 N-121328b_2_6-1-2 
##                58                46                25                52 
##    N-113674_A-1-2  S-127438_2_5-1-2  N-121342_2_7-1-2  N-121330_2_3-1-2 
##                 1                96                87                18 
##  N-121335_2_1-1-2  S-121338_2_2-1-2    S-121340_3-1-2  S-121321_2_2-1-2 
##                21                68                73                34 
##    S-127438_B-1-2  S-121338_2_3-1-2  N-121342_2_2-1-2   N-121328a_2-1-2 
##                97                69                84                48 
##  N-121341_2_2-1-2    N-121330_A-1-2 N-121328b_2_3-1-2  N-121330_2_4-1-2 
##                76                19                50                57 
##    N-121344_A-1-2   N-121329b_1-1-2  N-121335_2_3-1-2  S-121323_2_4-1-2 
##                30                15                62                43 
##  N-121341_2_7-1-2 N-121328b_2_4-1-2  N-121322_2_4-1-2    N-121342_1-1-2 
##                80                11                 6                82 
##  N-121342_2_3-1-2    N-121335_A-1-2   N-121329b_2-1-2  N-121330_2_2-1-2 
##                27                65                55                56 
##  N-121341_2_5-1-2  N-121342_2_5-1-2    S-121338_A-1-2   N-121328b_3-1-2 
##                79                85                24                12 
##   N-121329b_3-1-2  N-121335_2_4-1-2    S-121340_2-1-2   N-121328b_2-1-2 
##                16                63                72                53 
## N-121329a_2_2-1-2    N-121322_C-1-2  N-121322_2_1-1-2  N-121341_2_3-1-2 
##                14                39                36                77 
## N-121328b_2_1-1-2  S-121338_2_4-1-2  N-121342_2_4-1-2    N-121337_1-1-2 
##                10                70                28                67 
##    N-121322_A-1-2    N-121342_2-1-2    N-121344_B-1-2    S-121331_A-1-2 
##                37                88                31                59 
##    N-121322_B-1-2 
##                38
```

### 3.5.1 tps grid deformation from mean shape (specimen 94 = 127438\_2\_3)

**Shape difference between the mean reference shape and the most extreme outlier**

## 3.6 Shape space

```
## Importance of components:
##                            PC1     PC2     PC3     PC4     PC5     PC6      PC7
## Standard deviation     0.03098 0.01917 0.01330 0.01251 0.01132 0.01081 0.009671
## Proportion of Variance 0.35992 0.13778 0.06634 0.05871 0.04810 0.04384 0.035080
## Cumulative Proportion  0.35992 0.49771 0.56405 0.62276 0.67085 0.71469 0.749770
##                             PC8      PC9    PC10    PC11     PC12     PC13
## Standard deviation     0.008114 0.007911 0.00741 0.00704 0.006573 0.005907
## Proportion of Variance 0.024700 0.023480 0.02060 0.01859 0.016200 0.013090
## Cumulative Proportion  0.774470 0.797950 0.81854 0.83713 0.853340 0.866420
##                           PC14     PC15     PC16    PC17     PC18     PC19
## Standard deviation     0.00569 0.005472 0.005439 0.00517 0.004752 0.004489
## Proportion of Variance 0.01214 0.011230 0.011090 0.01003 0.008470 0.007560
## Cumulative Proportion  0.87857 0.889800 0.900890 0.91092 0.919390 0.926950
##                            PC20     PC21     PC22     PC23     PC24     PC25
## Standard deviation     0.004298 0.004102 0.003849 0.003677 0.003483 0.003411
## Proportion of Variance 0.006930 0.006310 0.005560 0.005070 0.004550 0.004360
## Cumulative Proportion  0.933880 0.940190 0.945750 0.950820 0.955370 0.959730
##                            PC26     PC27     PC28     PC29    PC30     PC31
## Standard deviation     0.003149 0.003044 0.002945 0.002779 0.00269 0.002499
## Proportion of Variance 0.003720 0.003470 0.003250 0.002900 0.00271 0.002340
## Cumulative Proportion  0.963450 0.966920 0.970180 0.973080 0.97579 0.978130
##                            PC32     PC33     PC34     PC35    PC36     PC37
## Standard deviation     0.002353 0.002275 0.002137 0.002038 0.00193 0.001848
## Proportion of Variance 0.002080 0.001940 0.001710 0.001560 0.00140 0.001280
## Cumulative Proportion  0.980210 0.982150 0.983860 0.985420 0.98682 0.988100
##                            PC38     PC39     PC40     PC41     PC42     PC43
## Standard deviation     0.001753 0.001669 0.001611 0.001522 0.001432 0.001386
## Proportion of Variance 0.001150 0.001040 0.000970 0.000870 0.000770 0.000720
## Cumulative Proportion  0.989250 0.990300 0.991270 0.992140 0.992910 0.993630
##                            PC44     PC45     PC46     PC47    PC48     PC49
## Standard deviation     0.001372 0.001333 0.001217 0.001196 0.00115 0.001092
## Proportion of Variance 0.000710 0.000670 0.000560 0.000540 0.00050 0.000450
## Cumulative Proportion  0.994330 0.995000 0.995560 0.996090 0.99659 0.997030
##                            PC50      PC51      PC52      PC53      PC54
## Standard deviation     0.001001 0.0009921 0.0009681 0.0009105 0.0008269
## Proportion of Variance 0.000380 0.0003700 0.0003500 0.0003100 0.0002600
## Cumulative Proportion  0.997410 0.9977800 0.9981300 0.9984400 0.9987000
##                             PC55      PC56      PC57      PC58      PC59
## Standard deviation     0.0007839 0.0007654 0.0007017 0.0006327 0.0006264
## Proportion of Variance 0.0002300 0.0002200 0.0001800 0.0001500 0.0001500
## Cumulative Proportion  0.9989300 0.9991500 0.9993300 0.9994800 0.9996300
##                             PC60      PC61      PC62      PC63      PC64
## Standard deviation     0.0005442 0.0004766 0.0004523 0.0003855 0.0003271
## Proportion of Variance 0.0001100 0.0000900 0.0000800 0.0000600 0.0000400
## Cumulative Proportion  0.9997400 0.9998300 0.9999000 0.9999600 1.0000000
##                             PC65      PC66      PC67      PC68
## Standard deviation     1.541e-16 4.659e-17 3.978e-17 2.221e-17
## Proportion of Variance 0.000e+00 0.000e+00 0.000e+00 0.000e+00
## Cumulative Proportion  1.000e+00 1.000e+00 1.000e+00 1.000e+00
```

### 3.6.1 Barplot indicating the variation described by principal components

**red line = mean variation**

### 3.6.2 Identification of number of meaningful principal components

```
##  [1] 3.599239e+01 1.377814e+01 6.634014e+00 5.871195e+00 4.809550e+00
##  [6] 4.383762e+00 3.508407e+00 2.469642e+00 2.347561e+00 2.059597e+00
## [11] 1.859116e+00 1.620318e+00 1.308742e+00 1.214358e+00 1.123153e+00
## [16] 1.109498e+00 1.002575e+00 8.469138e-01 7.559965e-01 6.929830e-01
## [21] 6.310440e-01 5.556051e-01 5.071157e-01 4.550547e-01 4.362914e-01
## [26] 3.719019e-01 3.474683e-01 3.254122e-01 2.897088e-01 2.714059e-01
## [31] 2.342685e-01 2.075943e-01 1.940816e-01 1.713022e-01 1.557679e-01
## [36] 1.397289e-01 1.281381e-01 1.152199e-01 1.044879e-01 9.735847e-02
## [41] 8.686846e-02 7.691620e-02 7.204905e-02 7.062027e-02 6.664237e-02
## [46] 5.555154e-02 5.361737e-02 4.962925e-02 4.473106e-02 3.757999e-02
## [51] 3.691656e-02 3.515385e-02 3.109769e-02 2.565041e-02 2.305087e-02
## [56] 2.197191e-02 1.846796e-02 1.501700e-02 1.471612e-02 1.110724e-02
## [61] 8.519045e-03 7.674144e-03 5.574018e-03 4.013736e-03 8.901576e-28
## [66] 8.142321e-29 5.936761e-29 1.849630e-29
```

```
## $tol
## [1] 1.333327
## 
## $good
## [1] 1 2
```

**number of meaningful principal components = 2**

### 3.6.3 PCA of shape variables

**North and south-basin gastropods**

**Sculpture groups L, I, S**

**DNA sequenced yes/no**

### 3.6.4 Lollipop plot of superimposed extreme shapes on PC1 and PC2

### 3.6.5 Contribution of landmarks on principal components (%)

```
##    LM PC1_contrib
## 16 16   10.647342
## 15 15    8.886241
## 17 17    8.585558
## 14 14    7.007648
## 32 32    6.726086
## 18 18    5.530259
## 30 30    4.689678
## 2   2    4.034081
## 26 26    4.002731
## 1   1    3.908778
## 31 31    3.849424
## 28 28    3.792523
## 27 27    3.730639
## 29 29    3.218969
## 4   4    3.064711
```

```
##    LM PC2_contrib
## 26 26   17.156301
## 21 21   13.566311
## 32 32    9.448825
## 6   6    7.114209
## 23 23    7.057566
## 31 31    6.334798
## 22 22    6.146152
## 11 11    5.396585
## 25 25    3.701181
```
